# Supplementary figures and images for: Molecular dissection of condensin II-mediated chromosome assembly using in vitro assays
Source: eLife. 2022 Aug 19;11:e78984. doi: 10.7554/eLife.78984 (PMC9433093; doi:10.7554/eLife.78984)

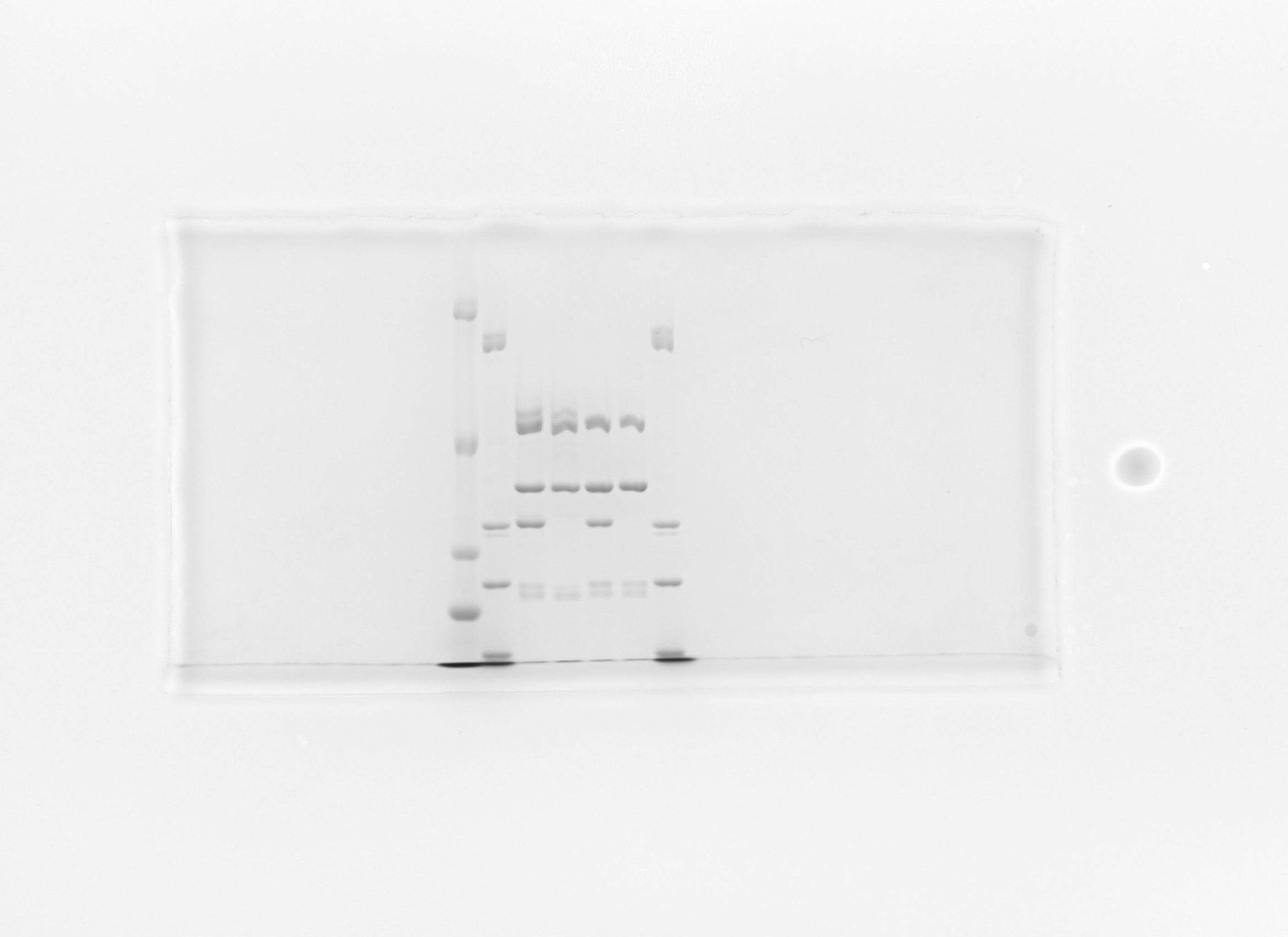

Supplement: Figure 1—figure supplement 1—source data 1. [file elife-78984-fig1-figsupp1-data1.zip › Figure 1-figure supplement 1-source data 1/Figure 1-figure supplement 1-source data 1-1A.tif]

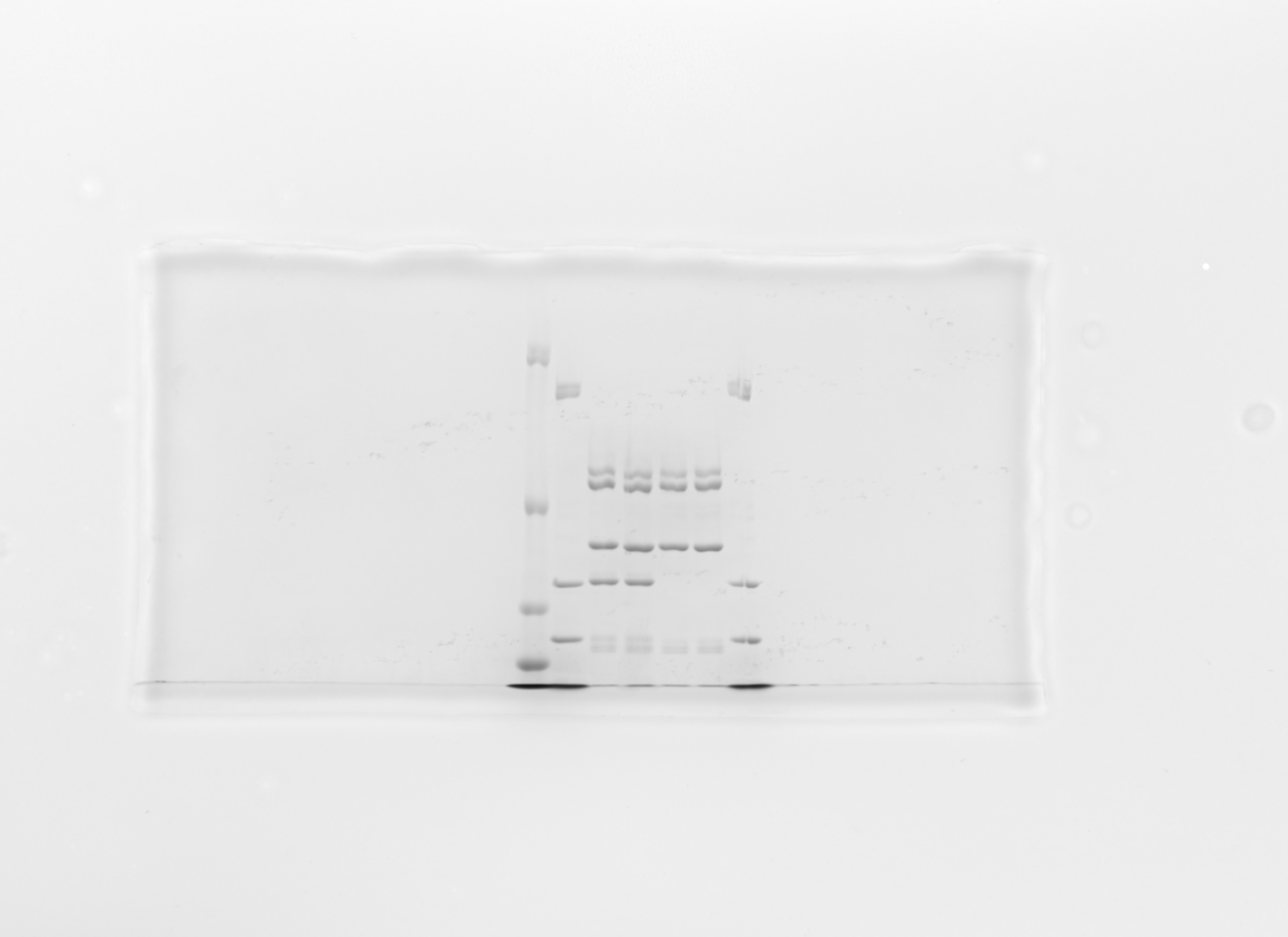

Supplement: Figure 1—figure supplement 1—source data 1. [file elife-78984-fig1-figsupp1-data1.zip › Figure 1-figure supplement 1-source data 1/Figure 1-figure supplement 1-source data 1-1B.tif]

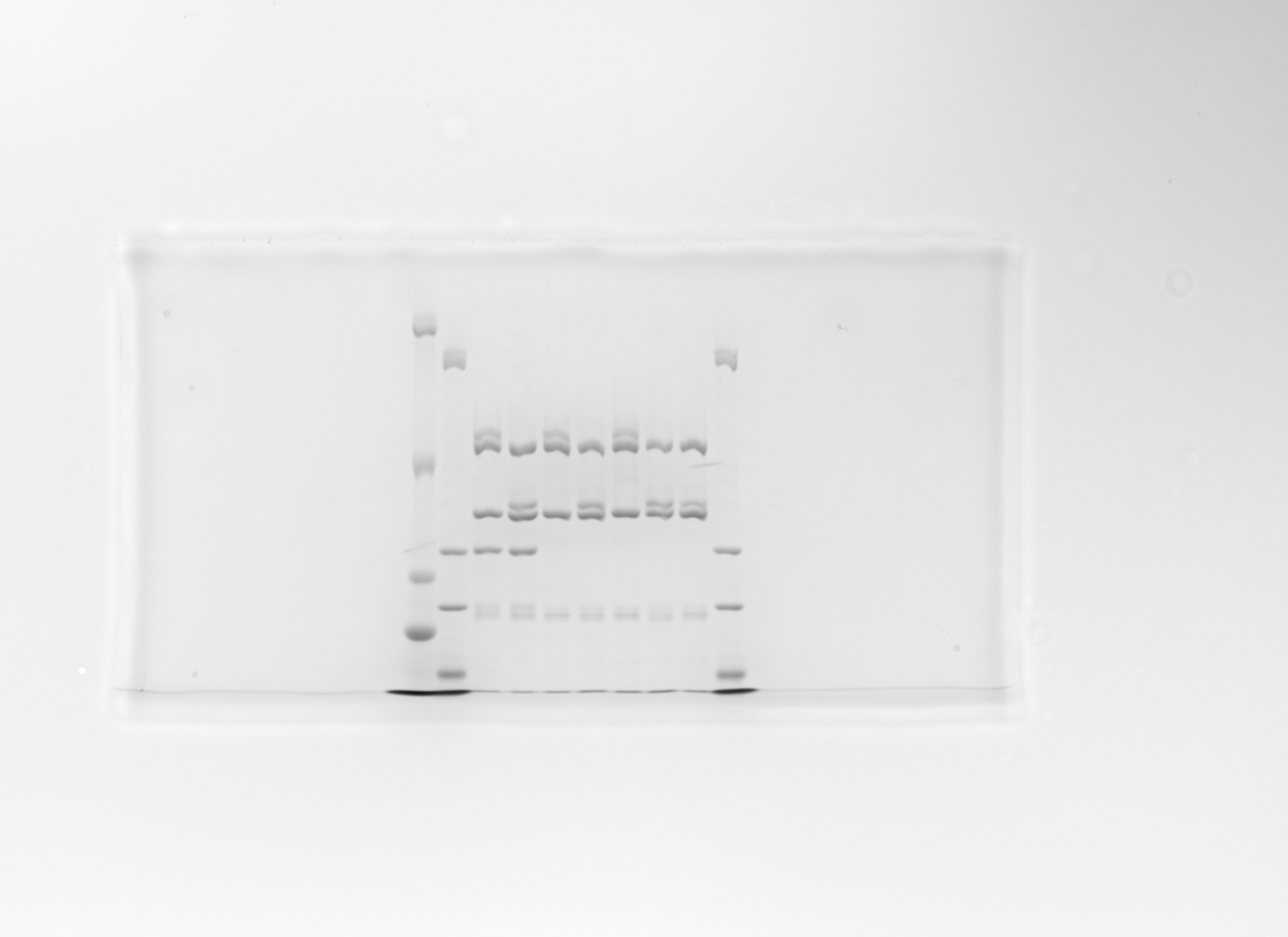

Supplement: Figure 1—figure supplement 1—source data 1. [file elife-78984-fig1-figsupp1-data1.zip › Figure 1-figure supplement 1-source data 1/Figure 1-figure supplement 1-source data 1-1C.tif]

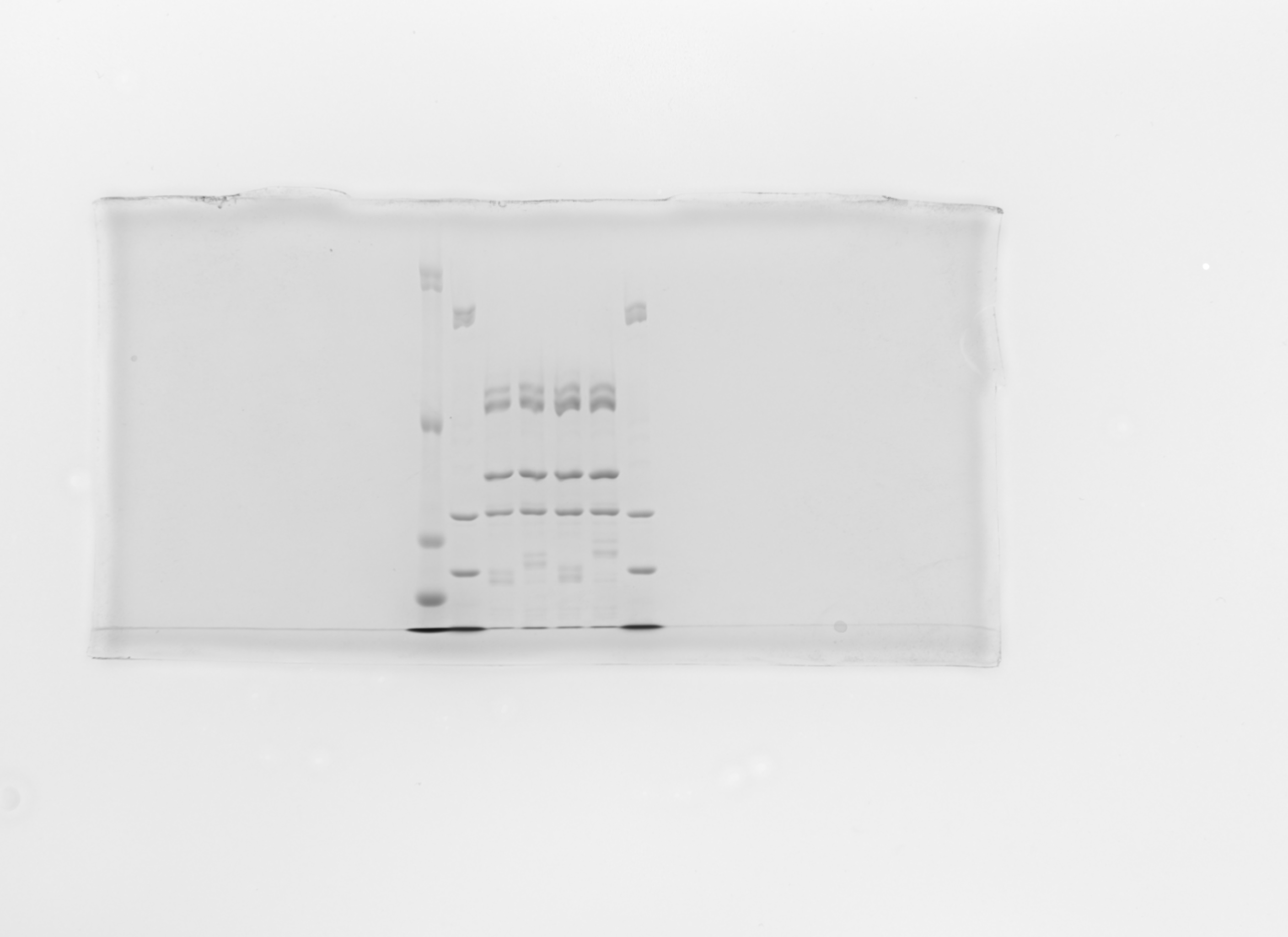

Supplement: Figure 1—figure supplement 1—source data 1. [file elife-78984-fig1-figsupp1-data1.zip › Figure 1-figure supplement 1-source data 1/Figure 1-figure supplement 1-source data 1-1D.tif]

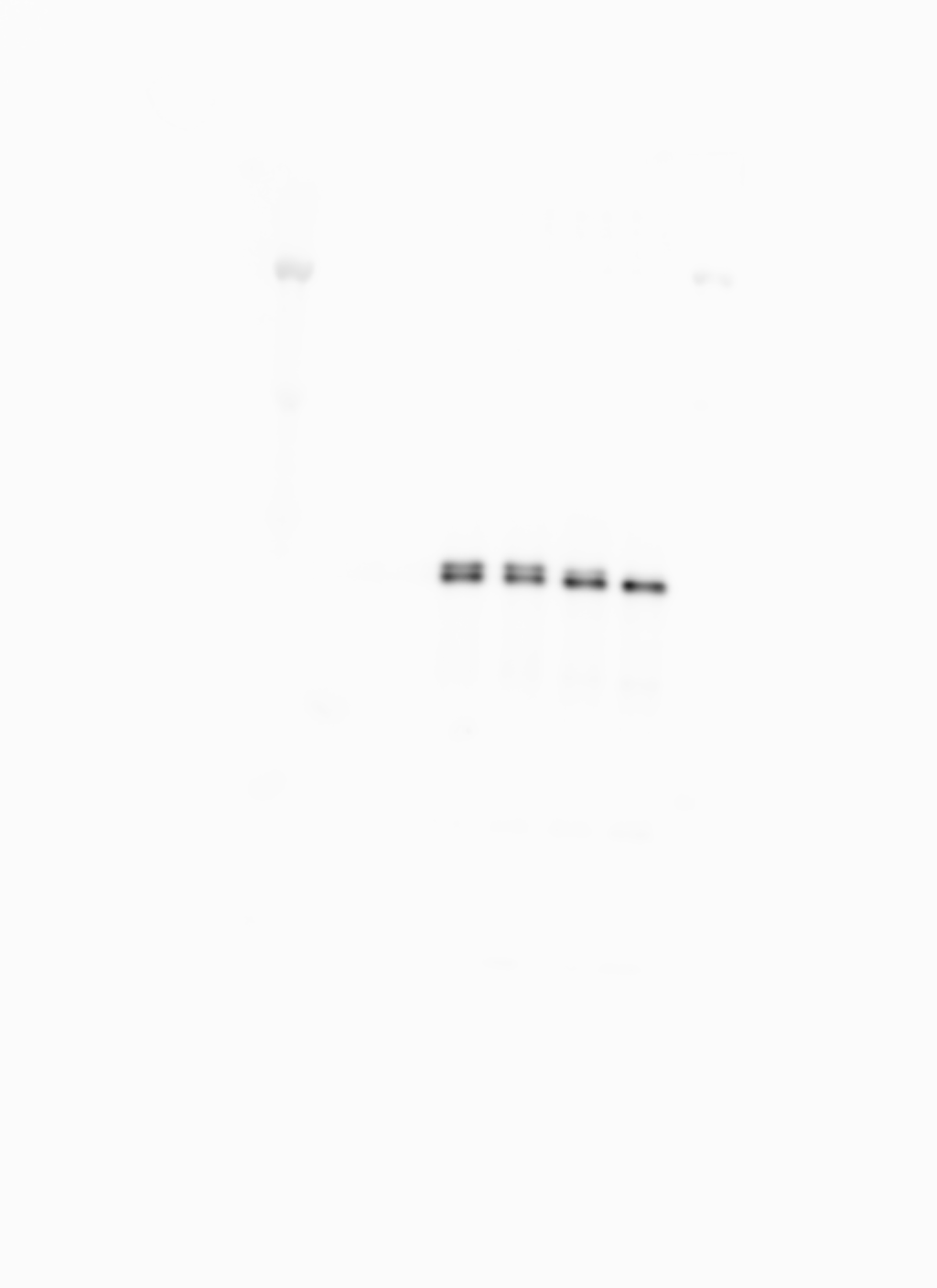

Supplement: Figure 1—figure supplement 1—source data 1. [file elife-78984-fig1-figsupp1-data1.zip › Figure 1-figure supplement 1-source data 1/Figure 1-figure supplement 1-source data 1-1E.tif]

Figure 1–figure supplement 1–source data 1

A

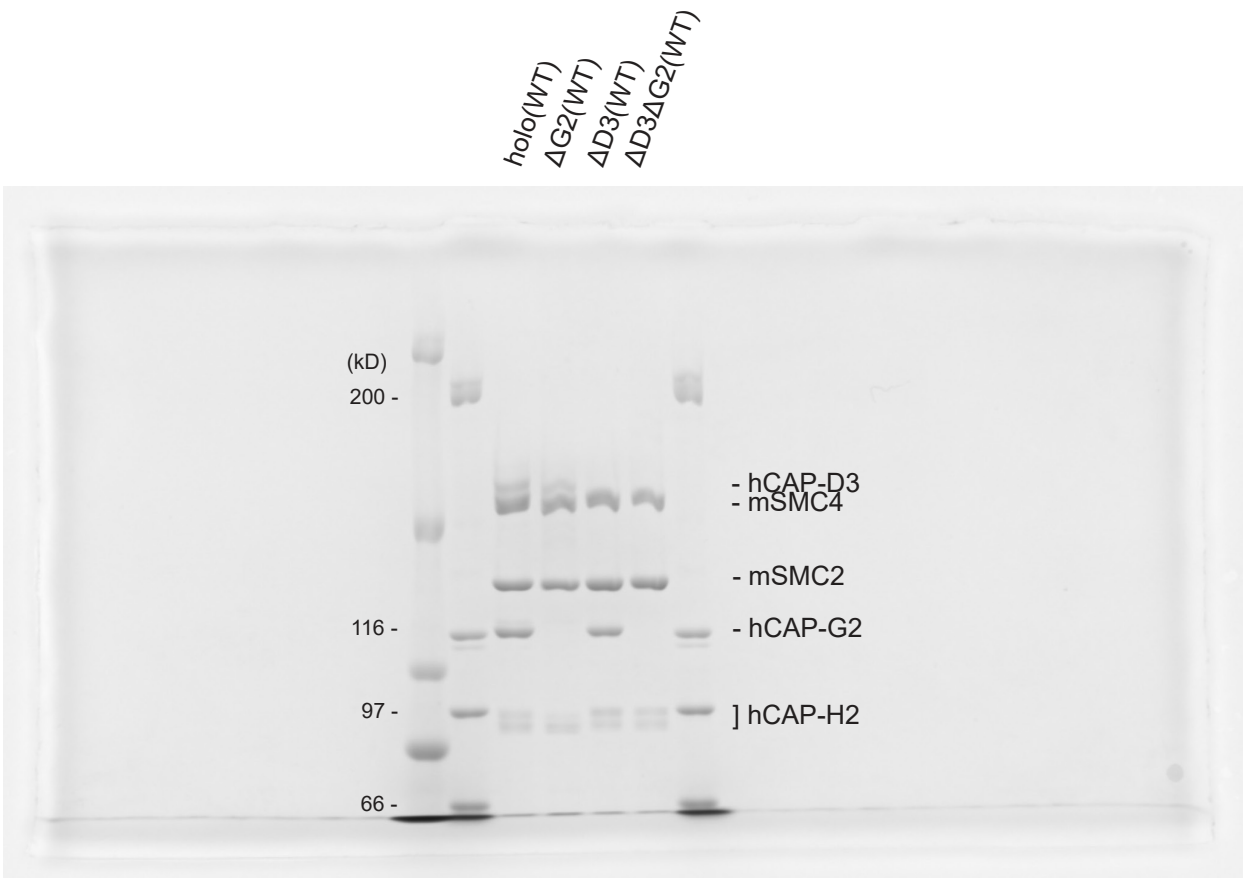

B

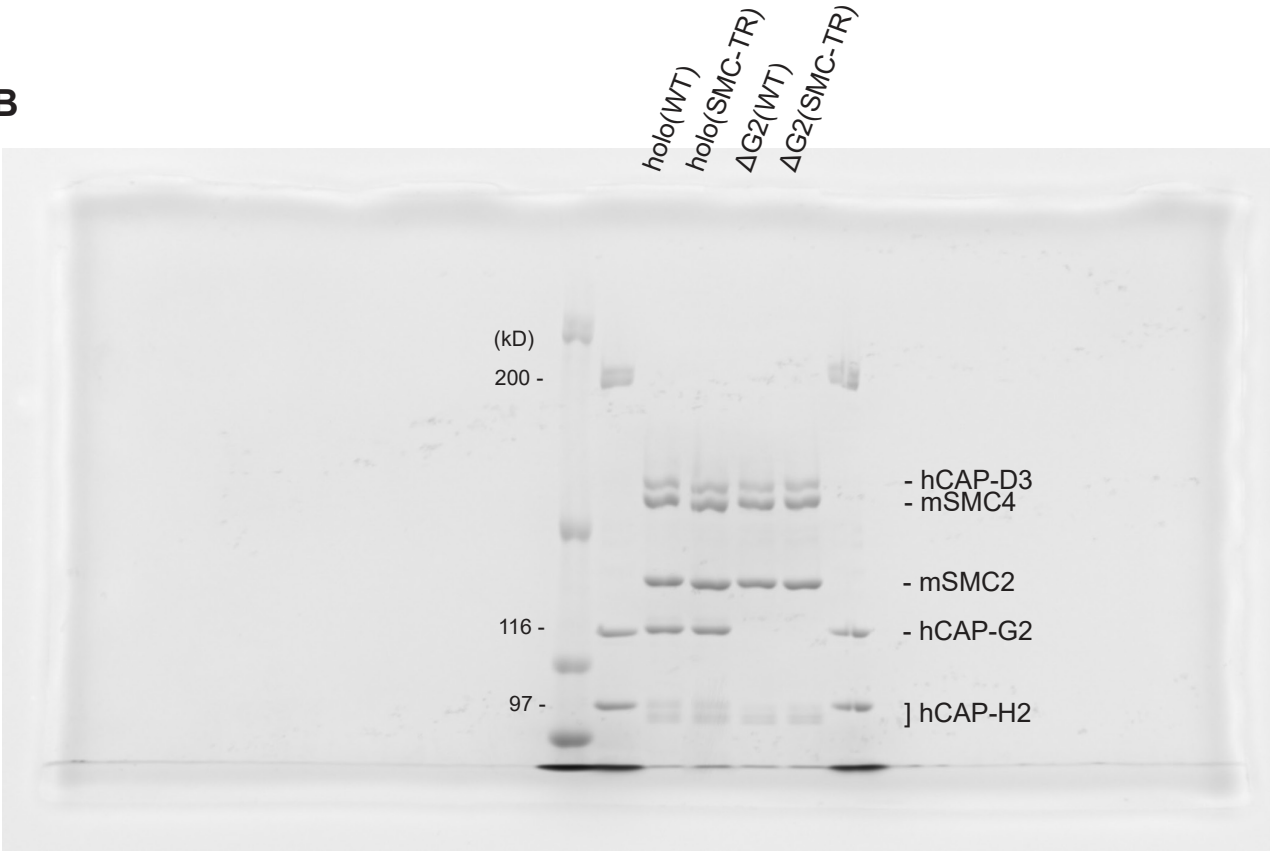

**C**

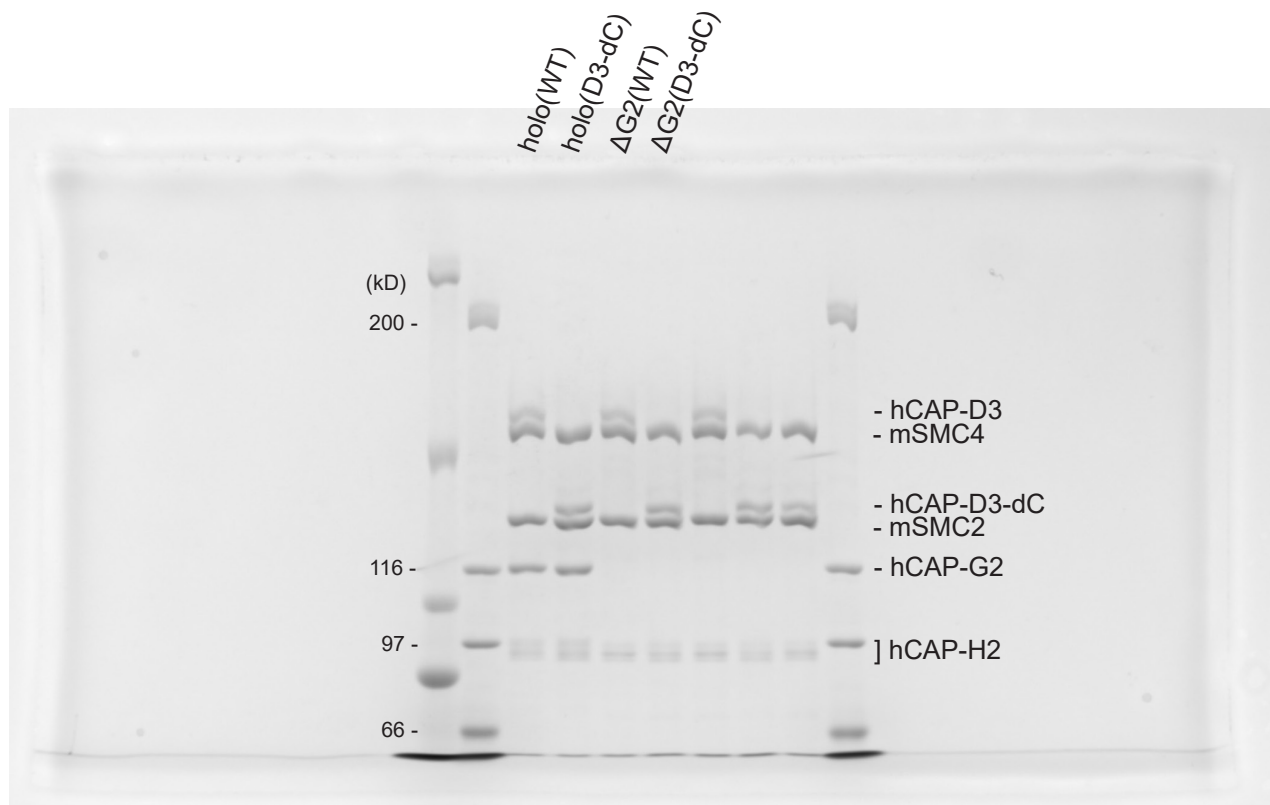

**D**

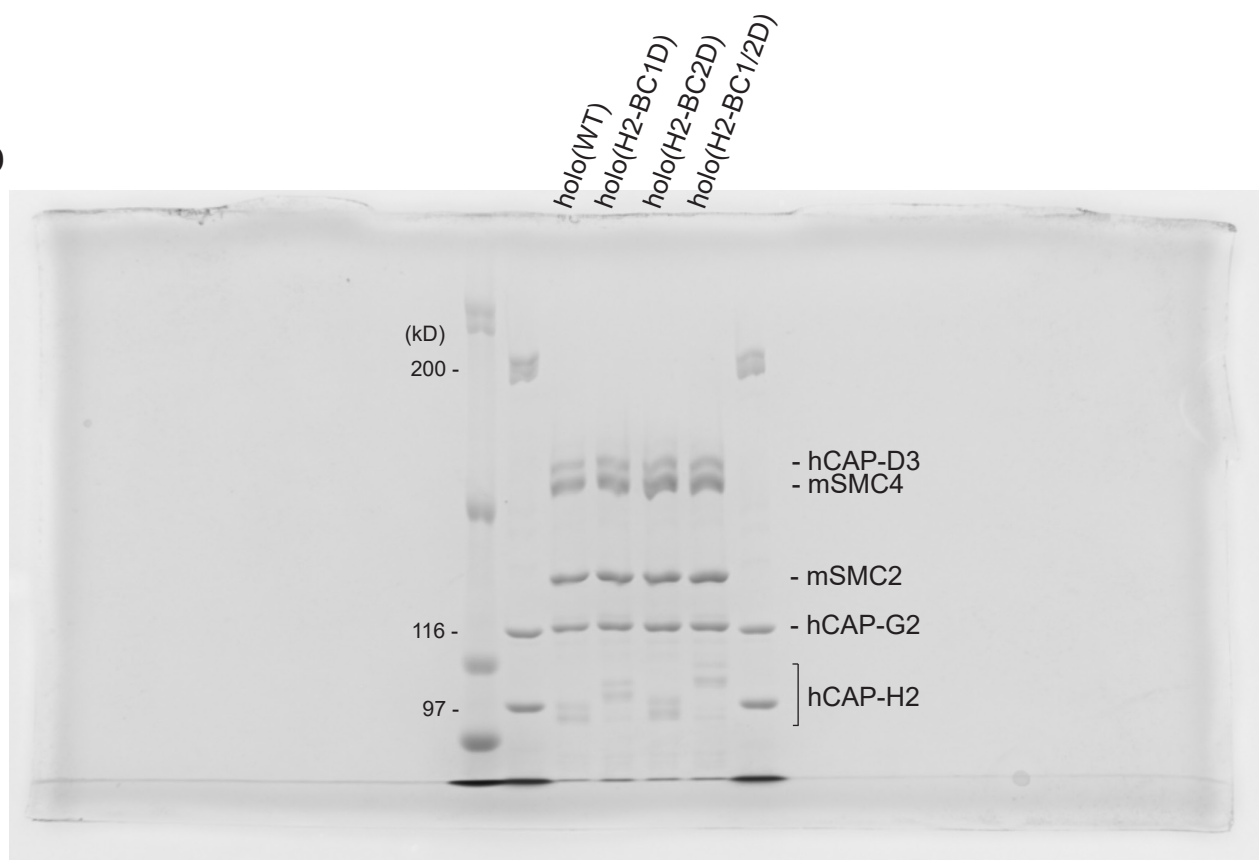

**E**

|           |   |   |   |   |   |   |
|-----------|---|---|---|---|---|---|
| holo(WT): | - | - | + | + | + | + |
| PPase:    | + | + | - | - | + | + |

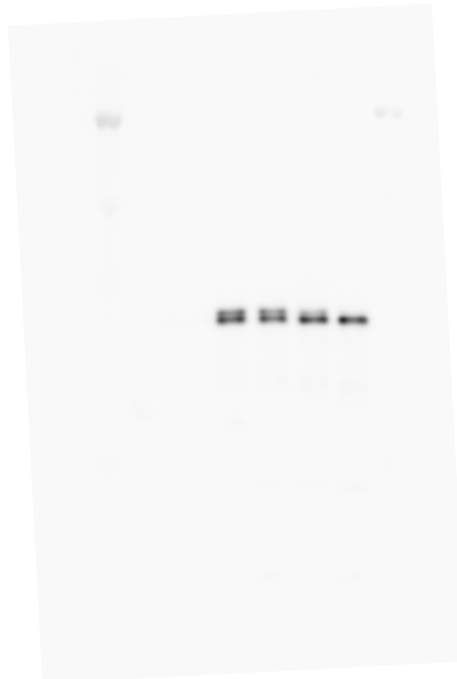

] hCAP-H2

Supplement: Figure 1—figure supplement 1—source data 1. [file elife-78984-fig1-figsupp1-data1.zip › Figure 1-figure supplement 1-source data 1/Figure 1-figure supplement 1-source data 1.pdf]

Figure 2–figure supplement 1–source data 1

A

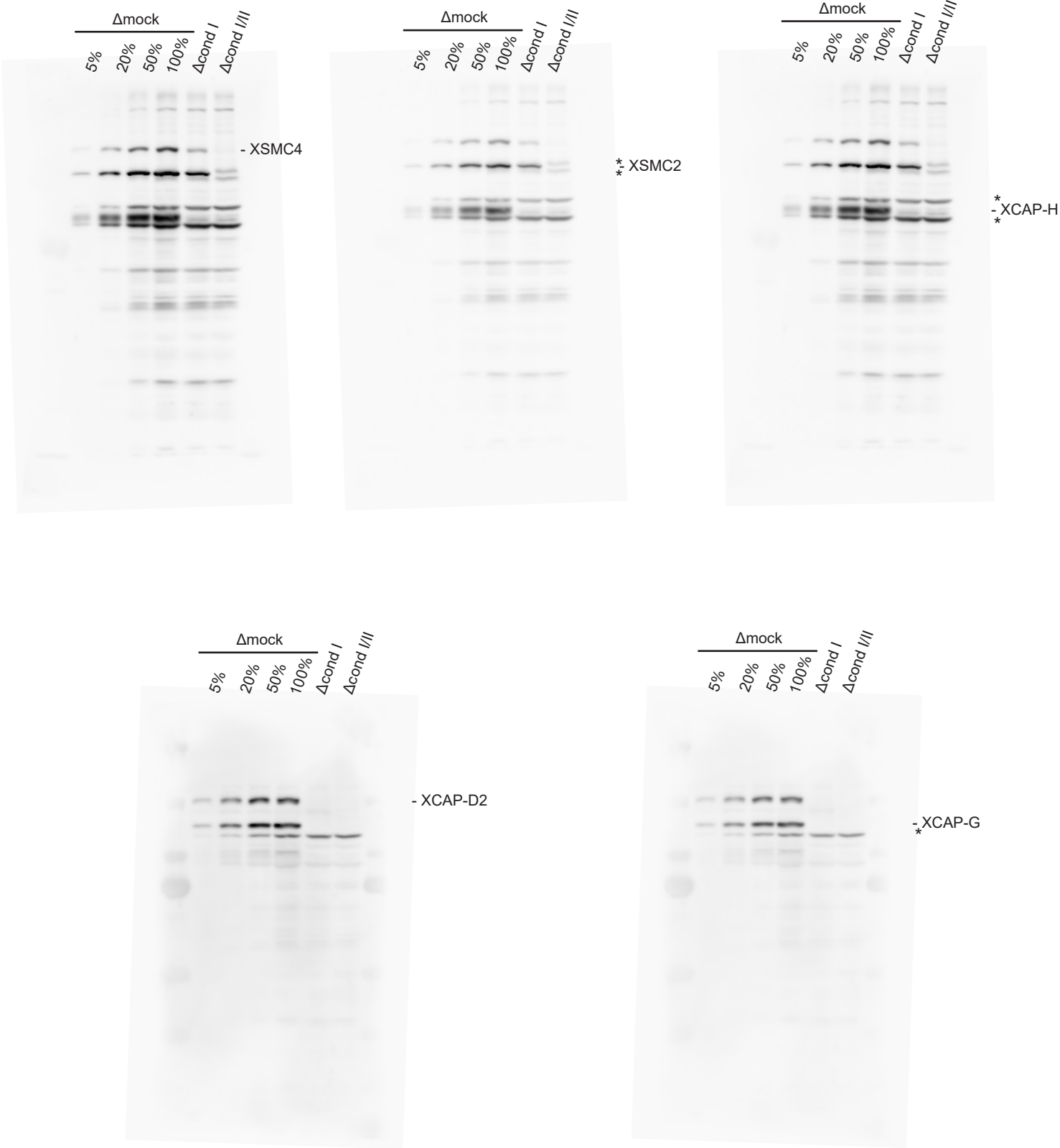

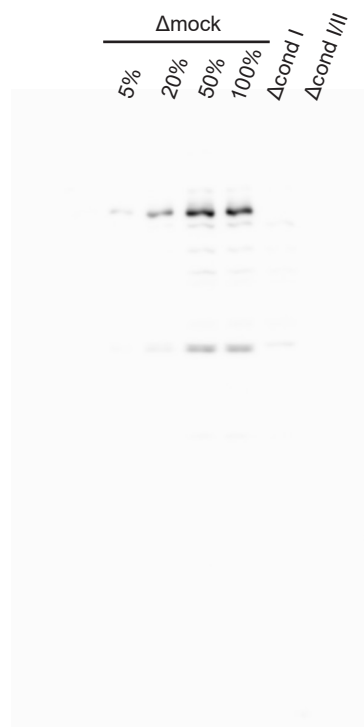

\* XCAP-D3

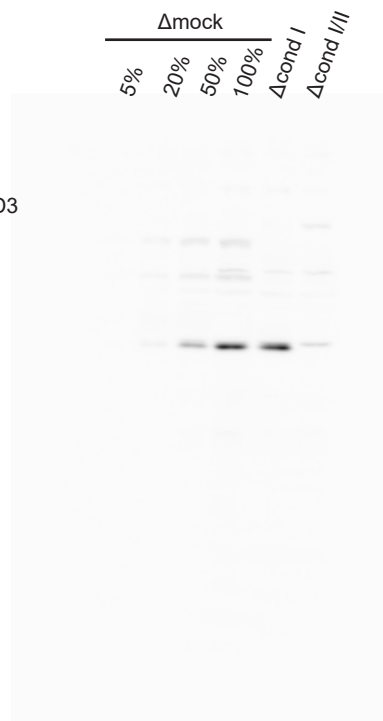

\* XCAP-H2  
(AfR201)

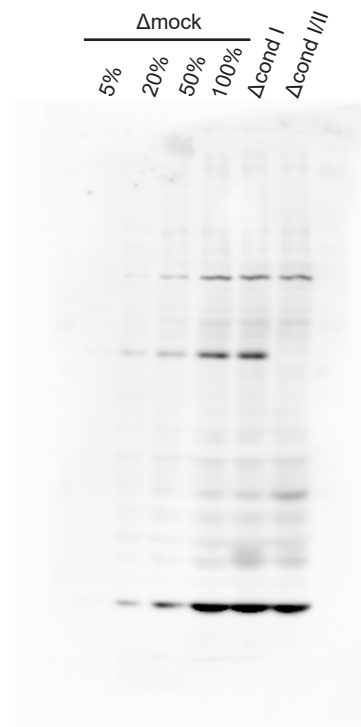

- XCAP-H2  
(AfR202)

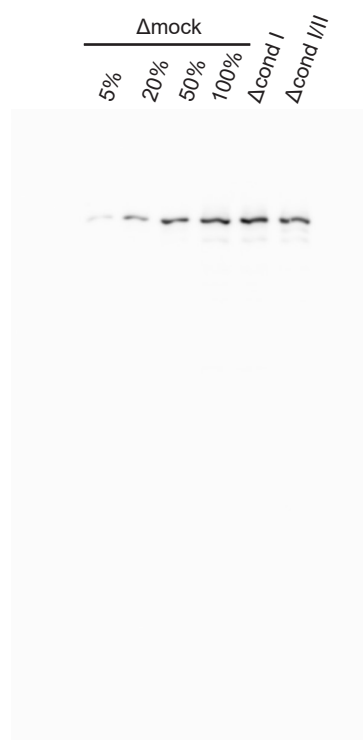

- Xtopo IIa

Supplement: Figure 2—figure supplement 1—source data 1. [file elife-78984-fig2-figsupp1-data1.zip › Figure 2-figure supplement 1-source data 1/Figure 2-figure supplement 1-source data 1.pdf]

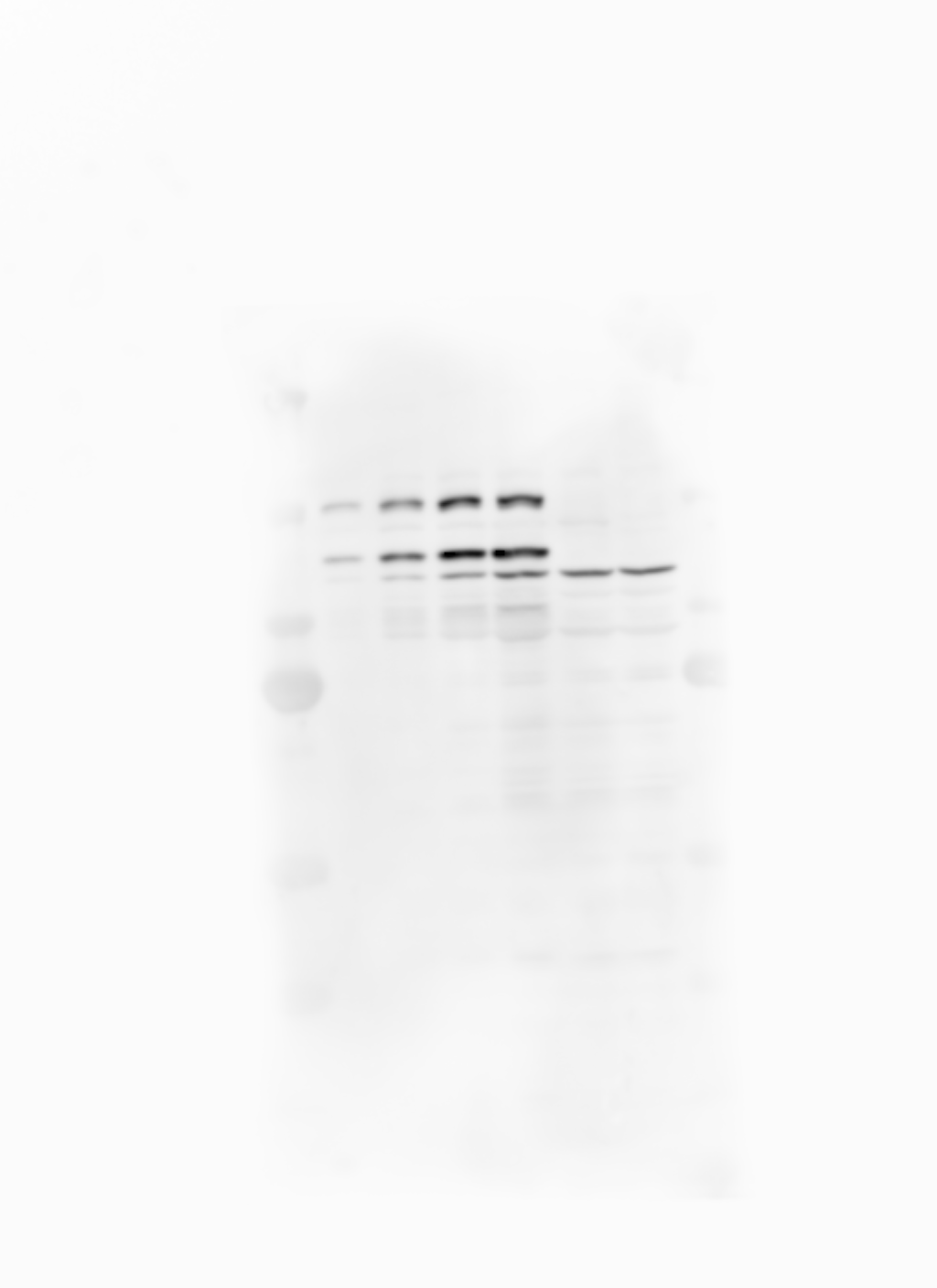

Supplement: Figure 2—figure supplement 1—source data 1. [file elife-78984-fig2-figsupp1-data1.zip › Figure 2-figure supplement 1-source data 1/Figure 2-figure supplement 1A-source data 1-XCAPD2.tif]

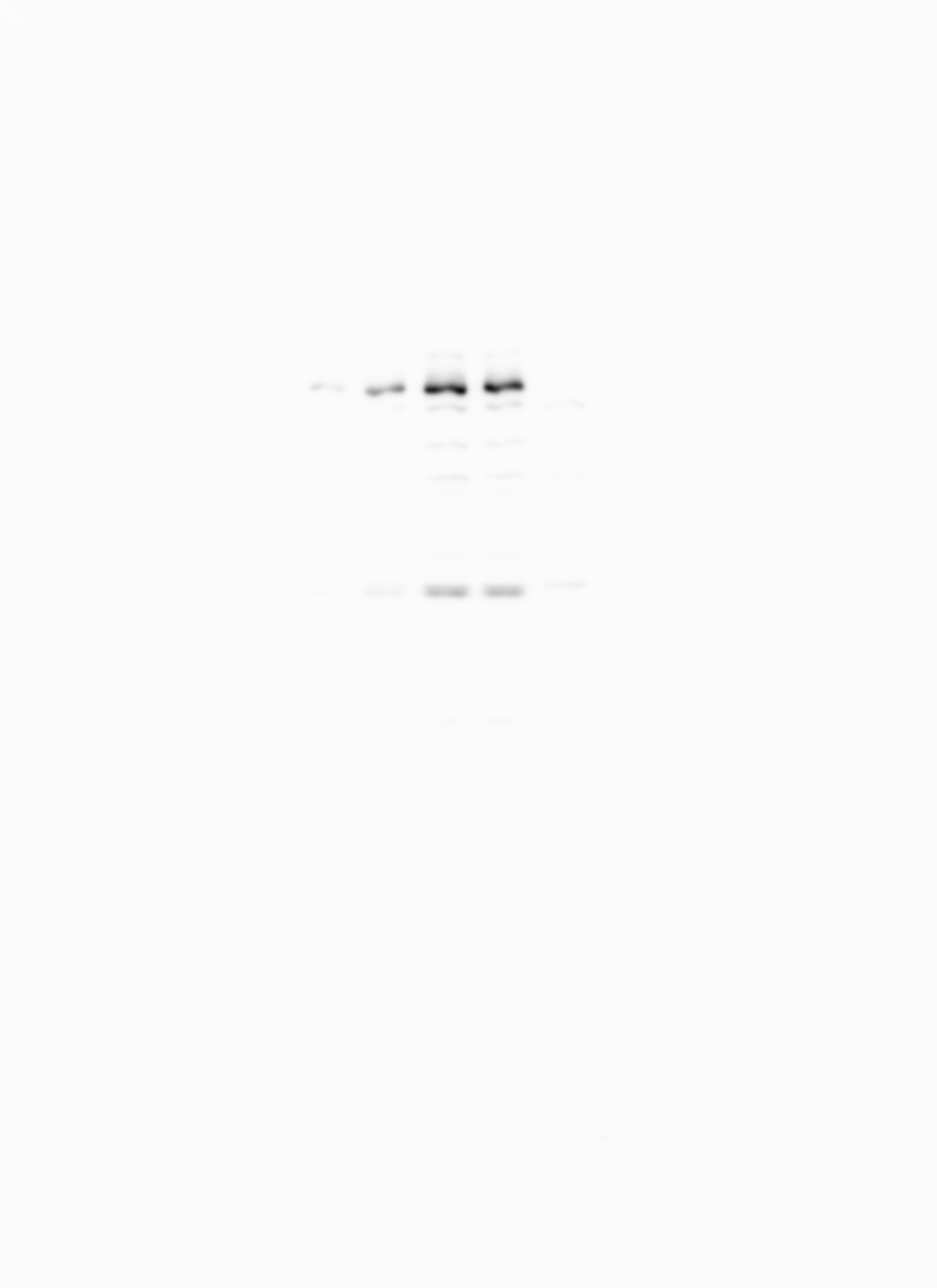

Supplement: Figure 2—figure supplement 1—source data 1. [file elife-78984-fig2-figsupp1-data1.zip › Figure 2-figure supplement 1-source data 1/Figure 2-figure supplement 1A-source data 1-XCAPD3.tif]

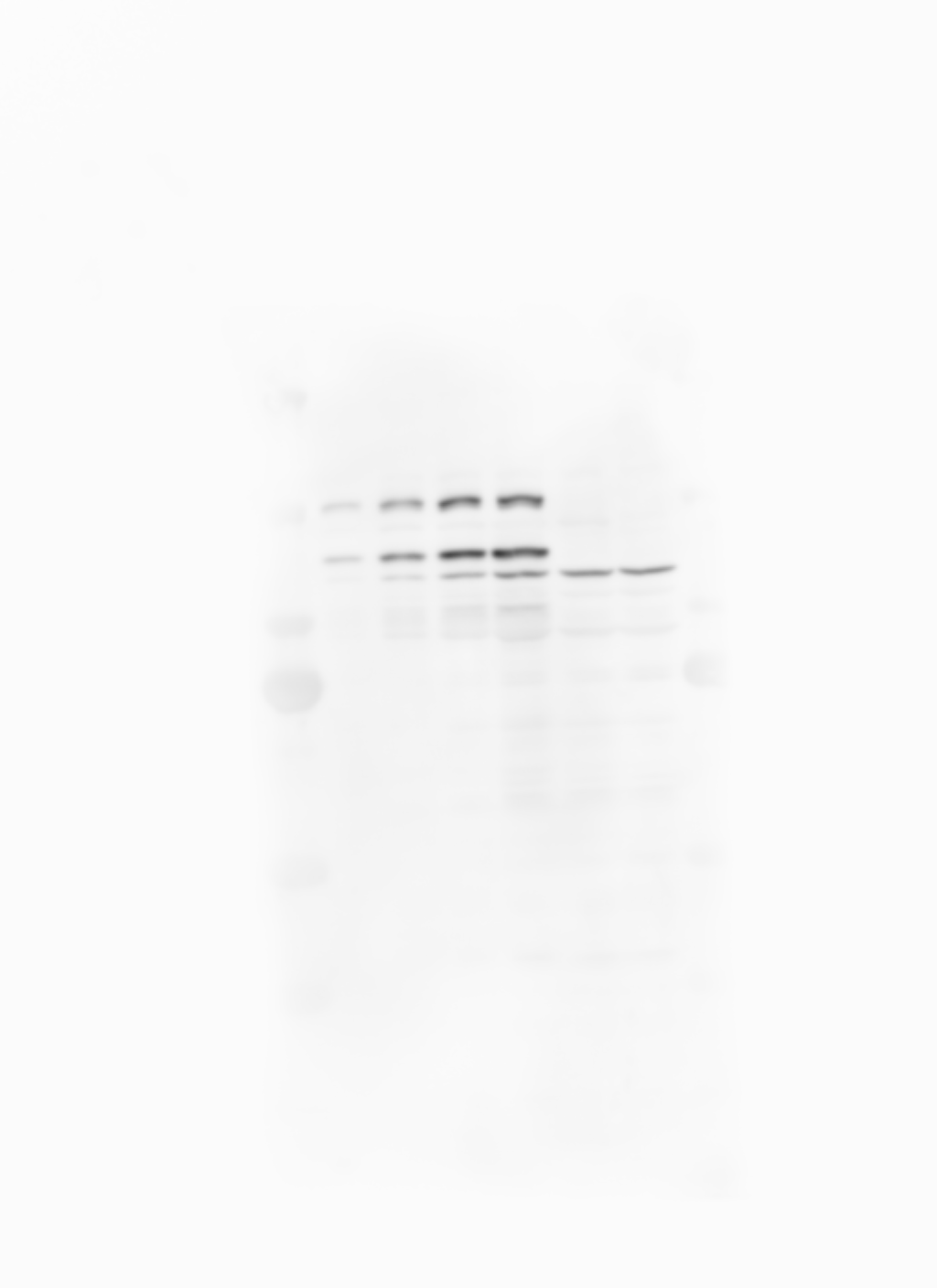

Supplement: Figure 2—figure supplement 1—source data 1. [file elife-78984-fig2-figsupp1-data1.zip › Figure 2-figure supplement 1-source data 1/Figure 2-figure supplement 1A-source data 1-XCAPG.tif]

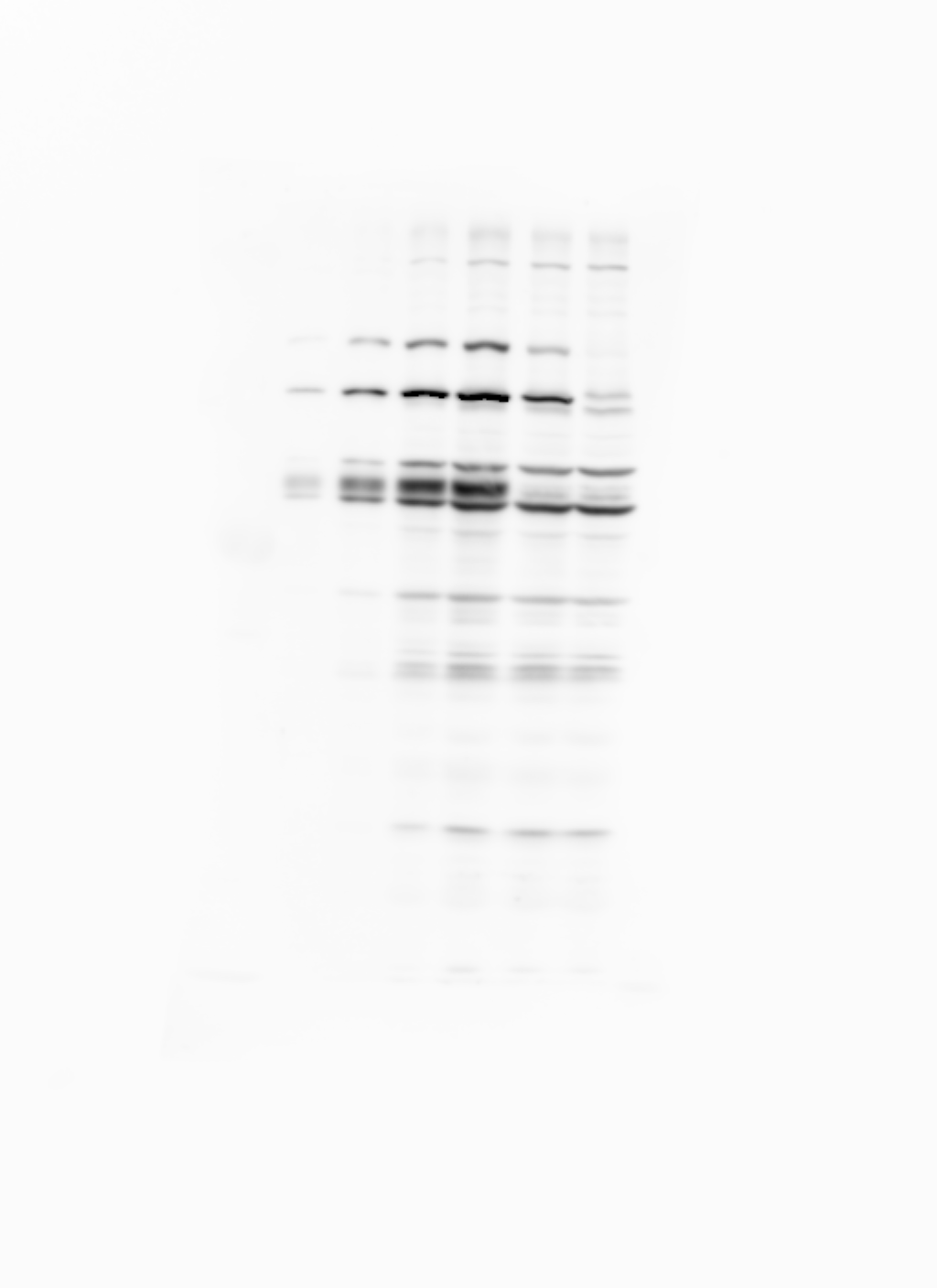

Supplement: Figure 2—figure supplement 1—source data 1. [file elife-78984-fig2-figsupp1-data1.zip › Figure 2-figure supplement 1-source data 1/Figure 2-figure supplement 1A-source data 1-XCAPH.tif]

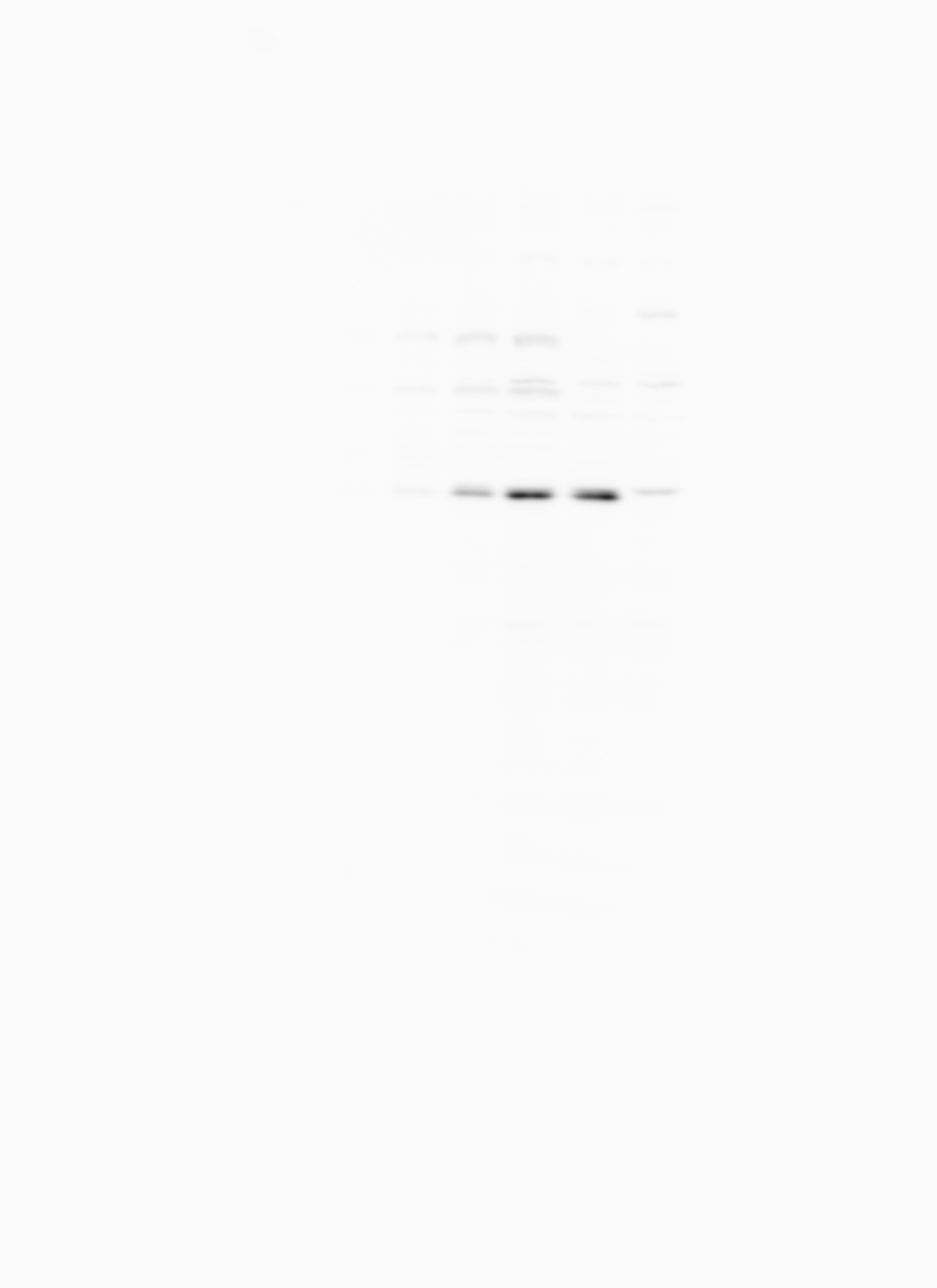

Supplement: Figure 2—figure supplement 1—source data 1. [file elife-78984-fig2-figsupp1-data1.zip › Figure 2-figure supplement 1-source data 1/Figure 2-figure supplement 1A-source data 1-XCAPH2 AfR201.tif]

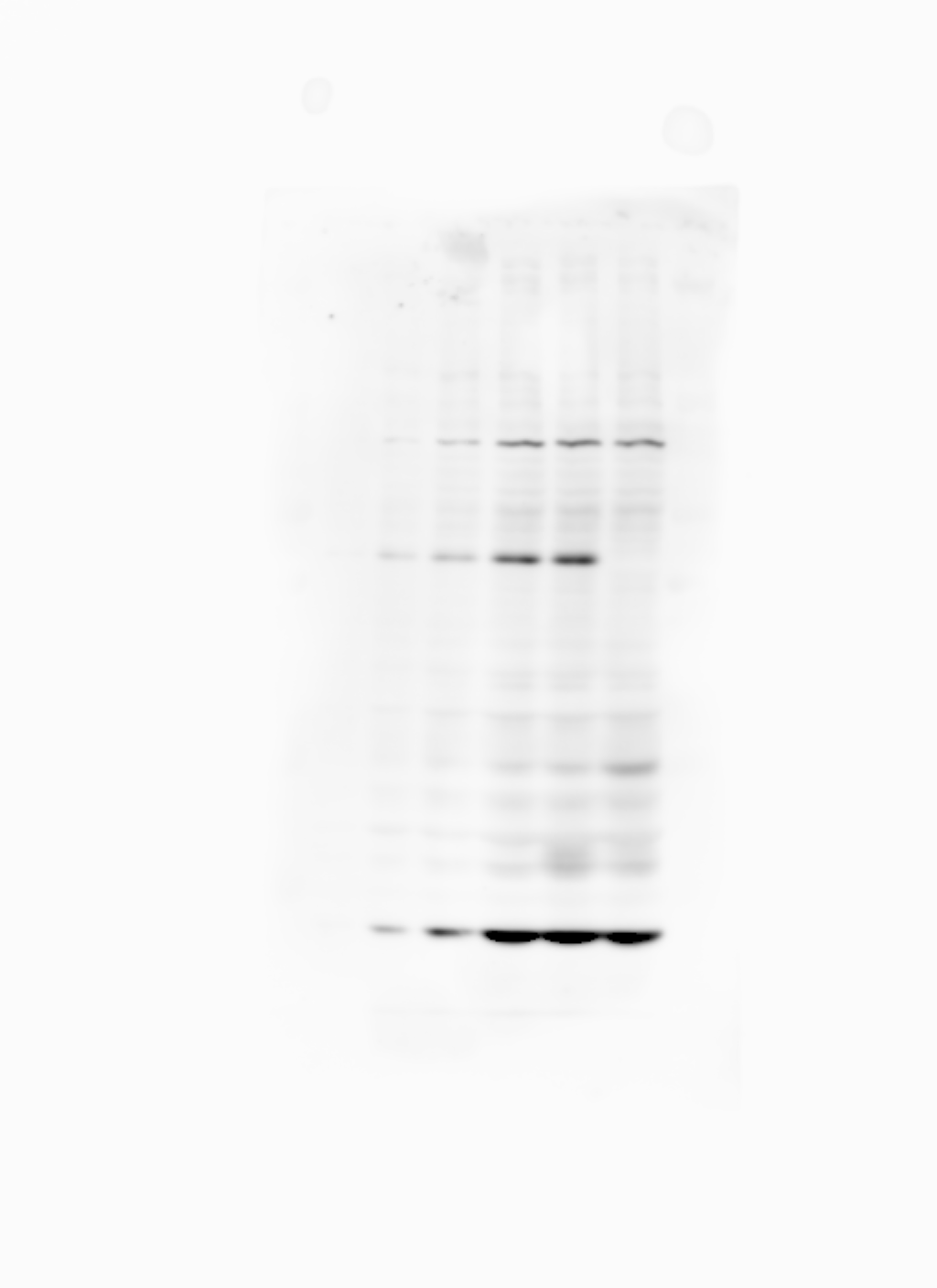

Supplement: Figure 2—figure supplement 1—source data 1. [file elife-78984-fig2-figsupp1-data1.zip › Figure 2-figure supplement 1-source data 1/Figure 2-figure supplement 1A-source data 1-XCAPH2 AfR202.tif]

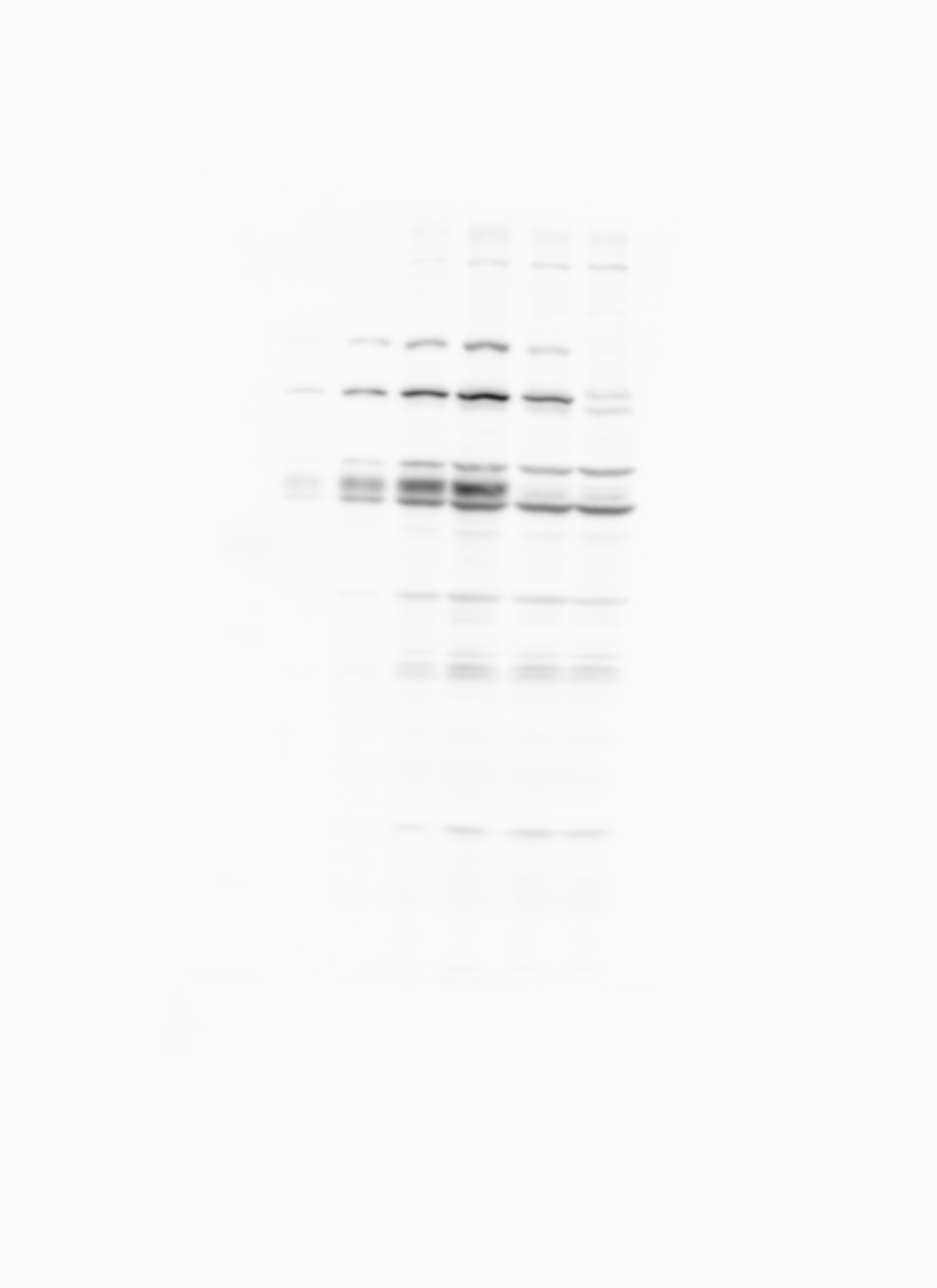

Supplement: Figure 2—figure supplement 1—source data 1. [file elife-78984-fig2-figsupp1-data1.zip › Figure 2-figure supplement 1-source data 1/Figure 2-figure supplement 1A-source data 1-XSMC2.tif]

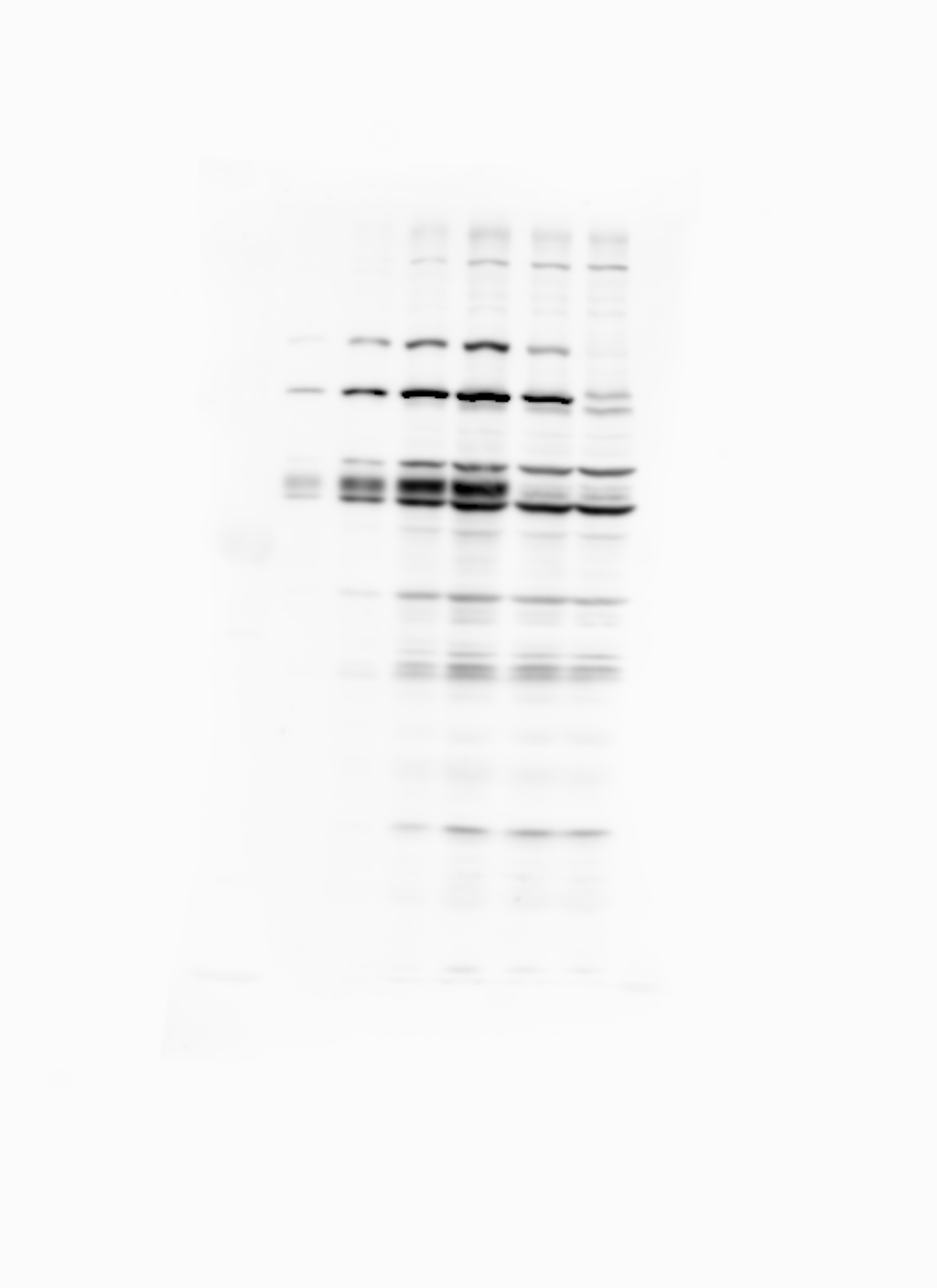

Supplement: Figure 2—figure supplement 1—source data 1. [file elife-78984-fig2-figsupp1-data1.zip › Figure 2-figure supplement 1-source data 1/Figure 2-figure supplement 1A-source data 1-XSMC4.tif]

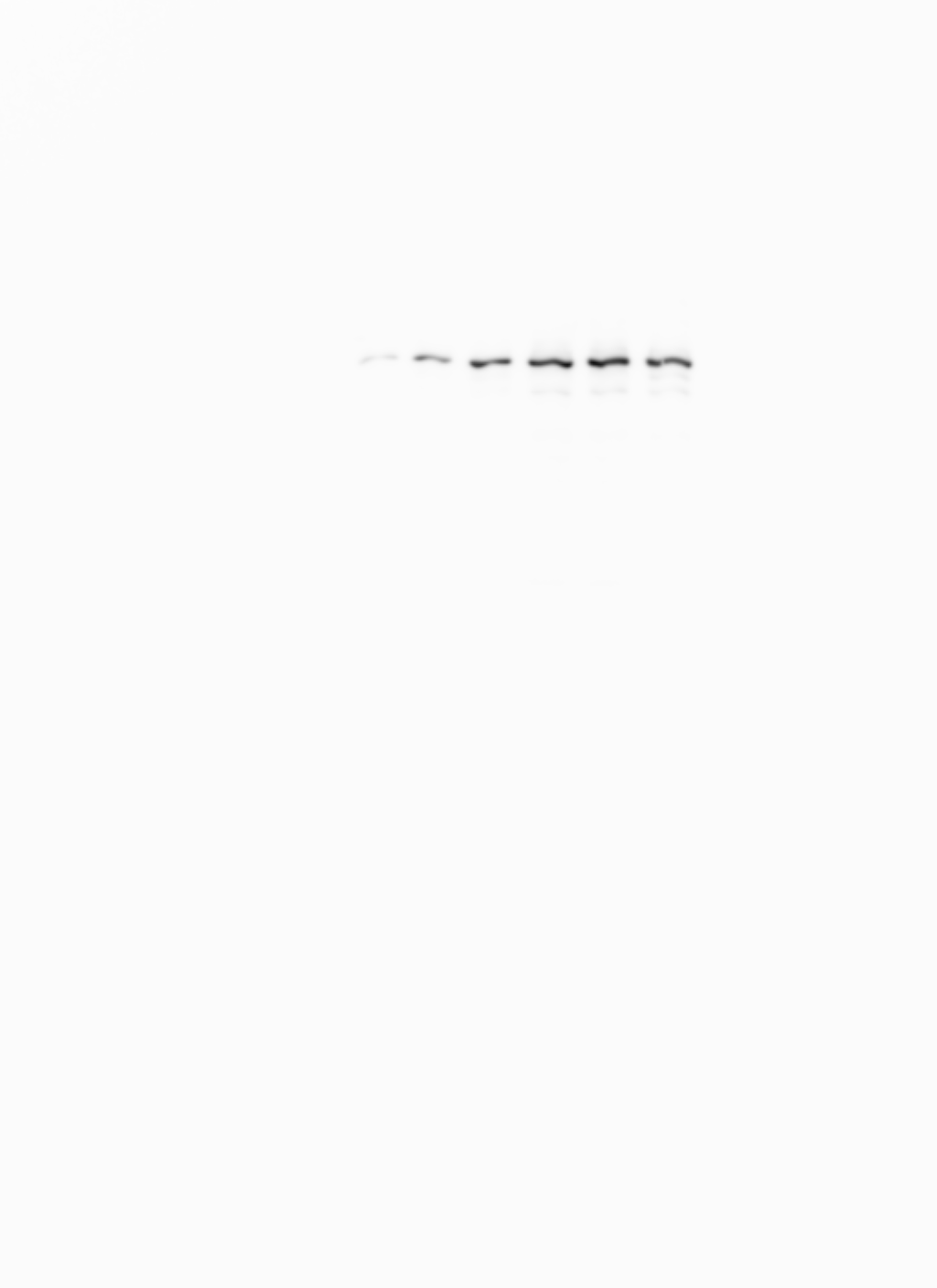

Supplement: Figure 2—figure supplement 1—source data 1. [file elife-78984-fig2-figsupp1-data1.zip › Figure 2-figure supplement 1-source data 1/Figure 2-figure supplement 1A-source data 1-Xtopo IIa.tif]

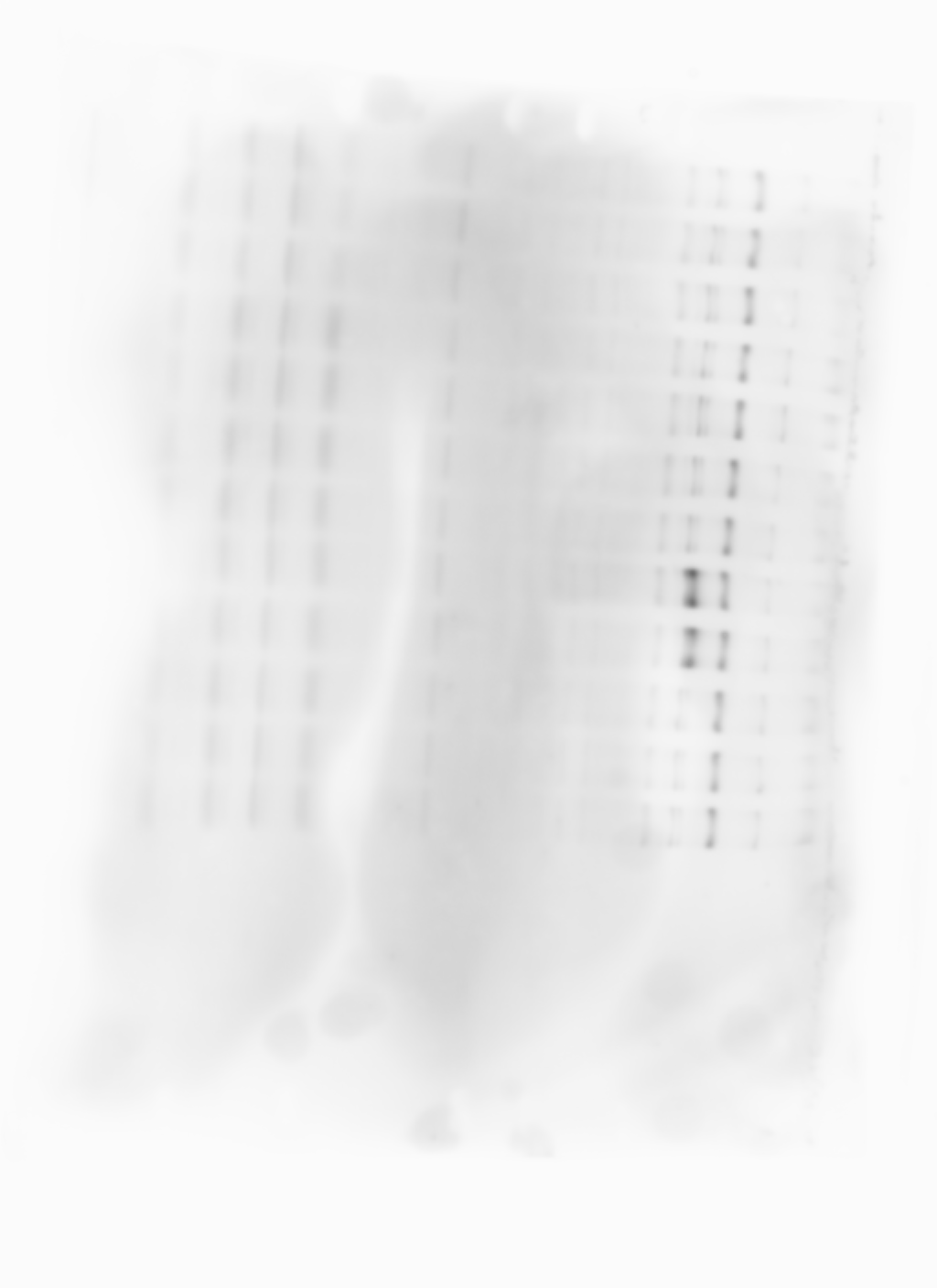

Supplement: Figure 4—figure supplement 1—source data 1. [file elife-78984-fig4-figsupp1-data1.zip › Figure 4-figure supplement 1-source data 1/Figure 4-figure suppelment 1B-source data 1-pT1415.tif]

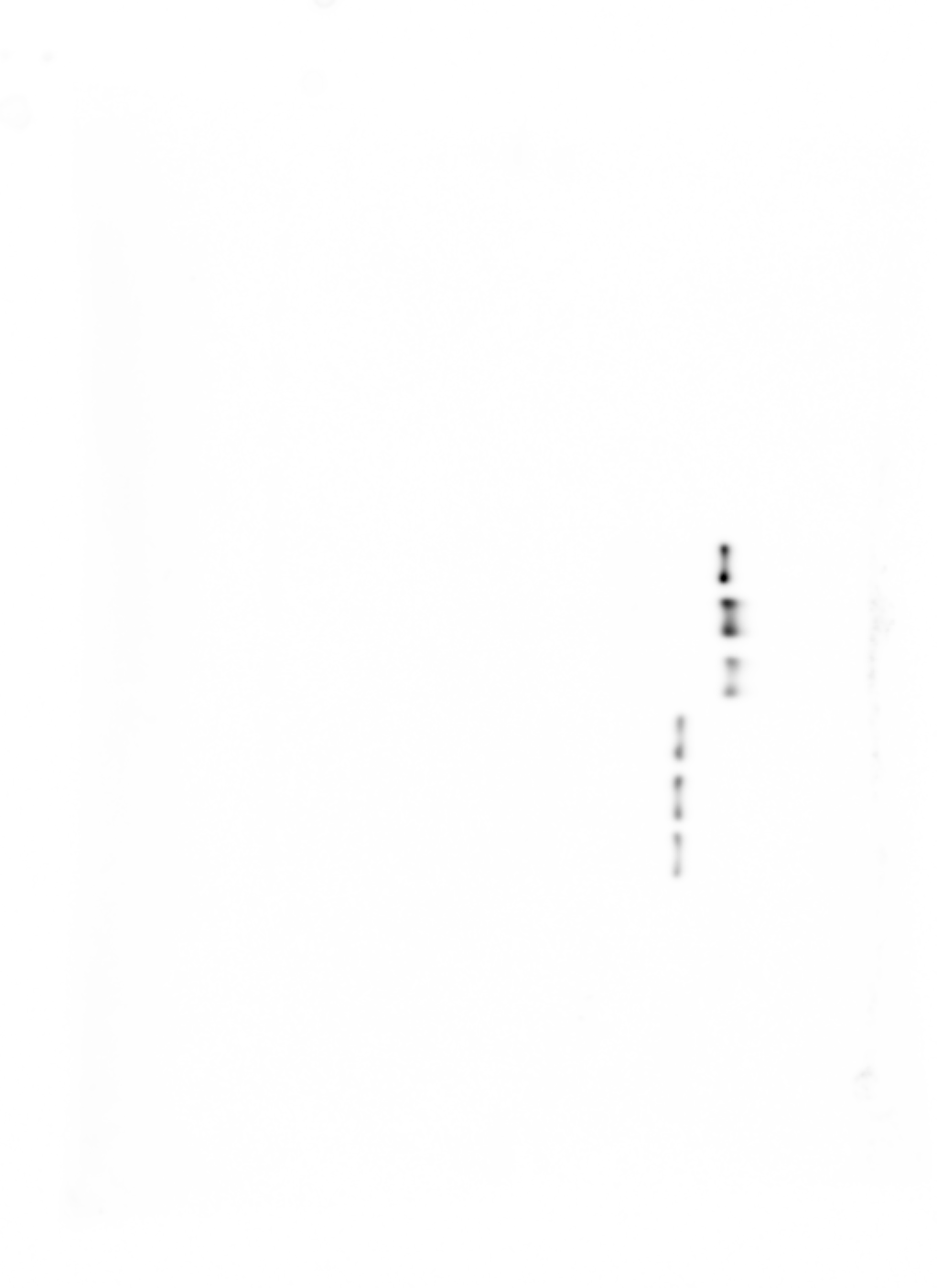

Supplement: Figure 4—figure supplement 1—source data 1. [file elife-78984-fig4-figsupp1-data1.zip › Figure 4-figure supplement 1-source data 1/Figure 4-figure supplement 1B-source data 1-hCAPD3.tif]

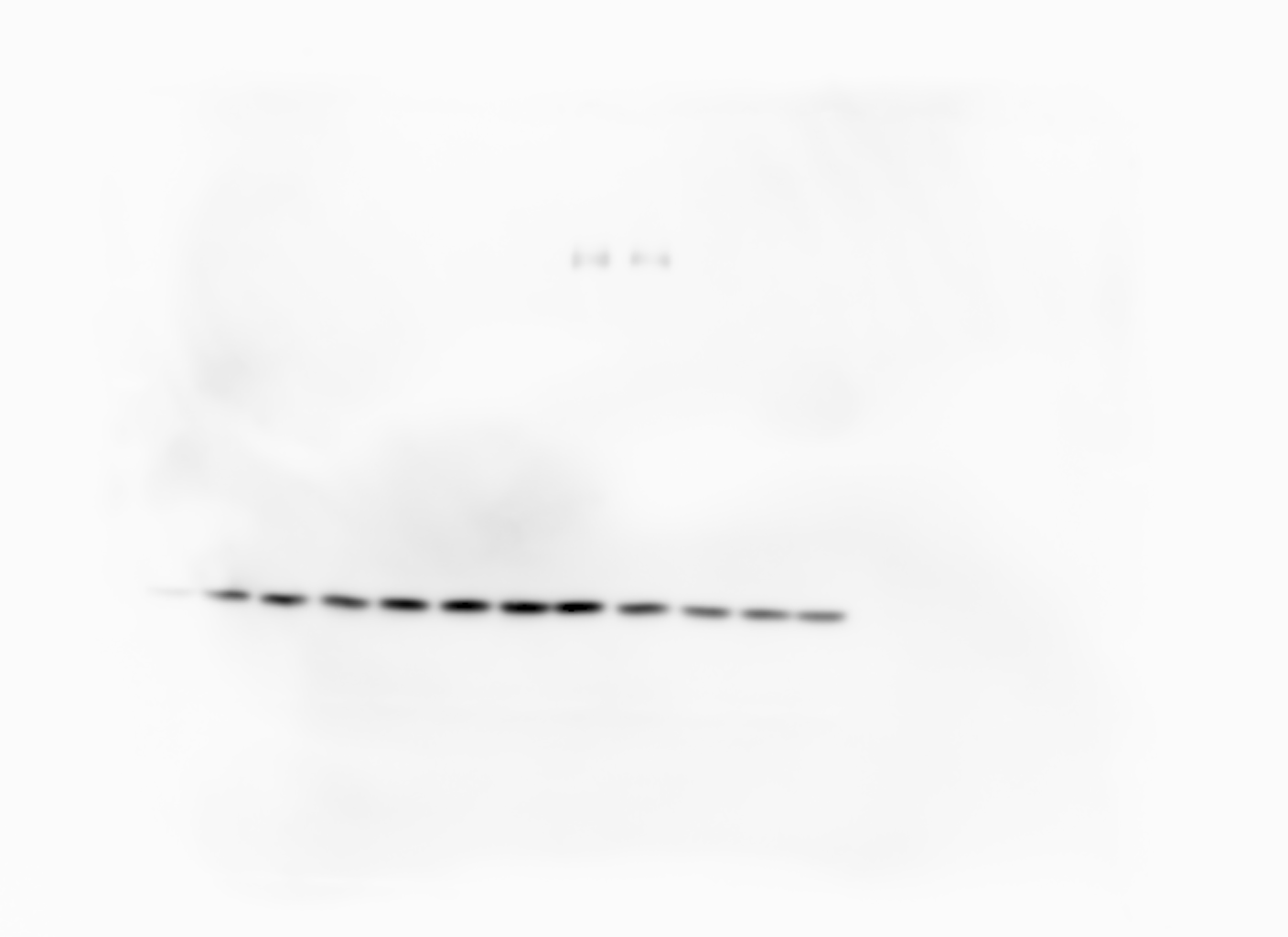

Supplement: Figure 4—figure supplement 1—source data 1. [file elife-78984-fig4-figsupp1-data1.zip › Figure 4-figure supplement 1-source data 1/Figure 4-figure supplement 1B-source data 1-pS1474.tif]

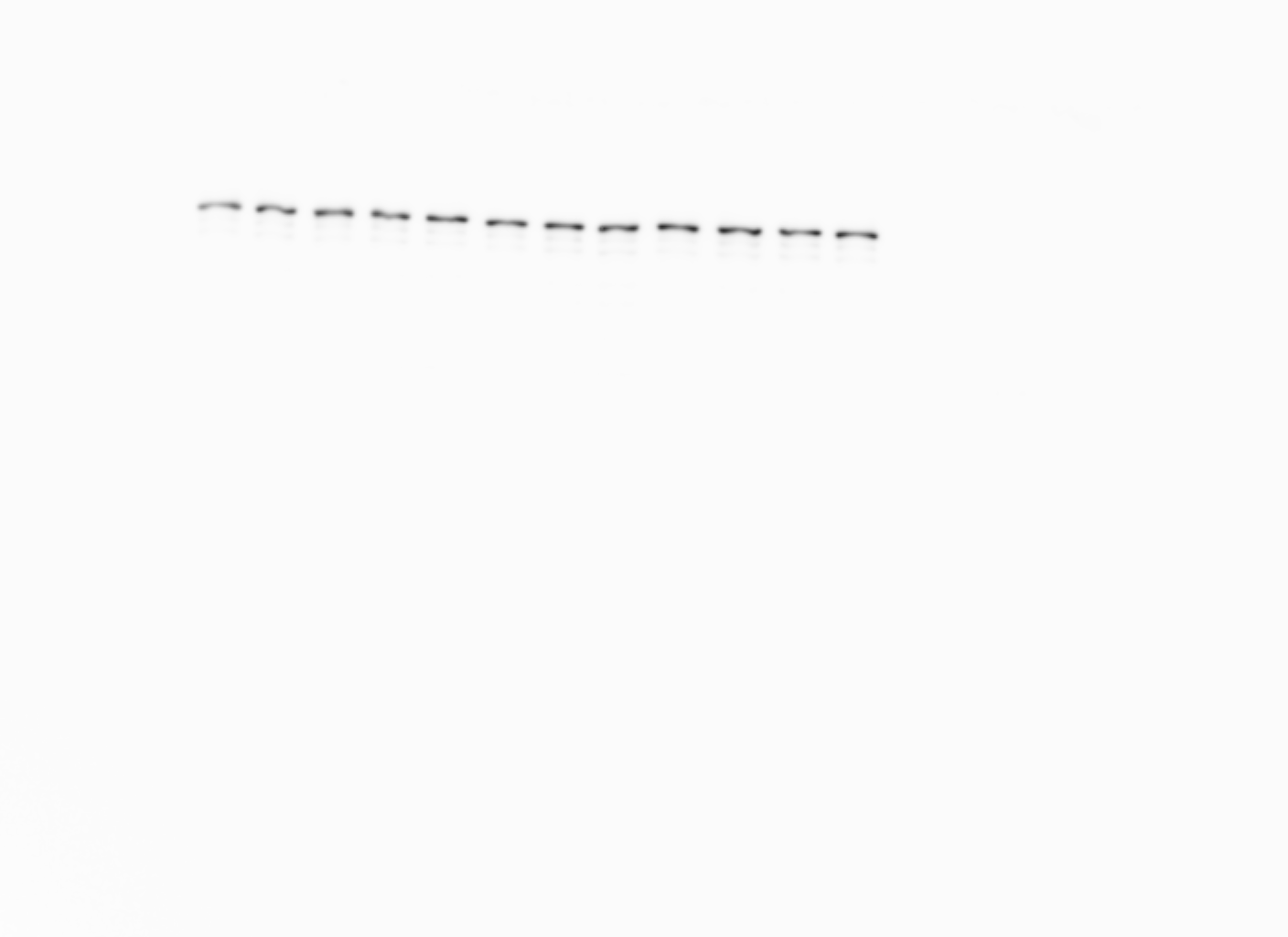

Supplement: Figure 4—figure supplement 1—source data 1. [file elife-78984-fig4-figsupp1-data1.zip › Figure 4-figure supplement 1-source data 1/Figure 4-figure supplement 1B-source data 1-Xtopo IIa.tif]

Figure 4–figure supplement 3B–source data 1

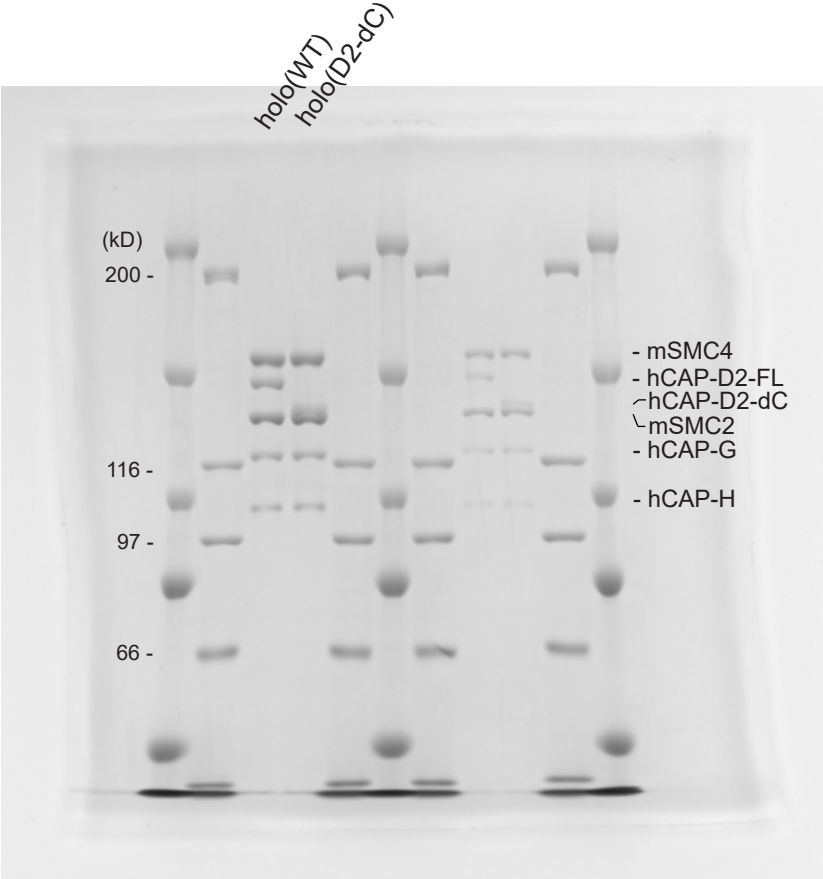

Supplement: Figure 4—figure supplement 3—source data 1. [file elife-78984-fig4-figsupp3-data1.zip › Figure 4-figure supplement 3-source data 1/Figure 4-figure supplement 3-source data 1.pdf]

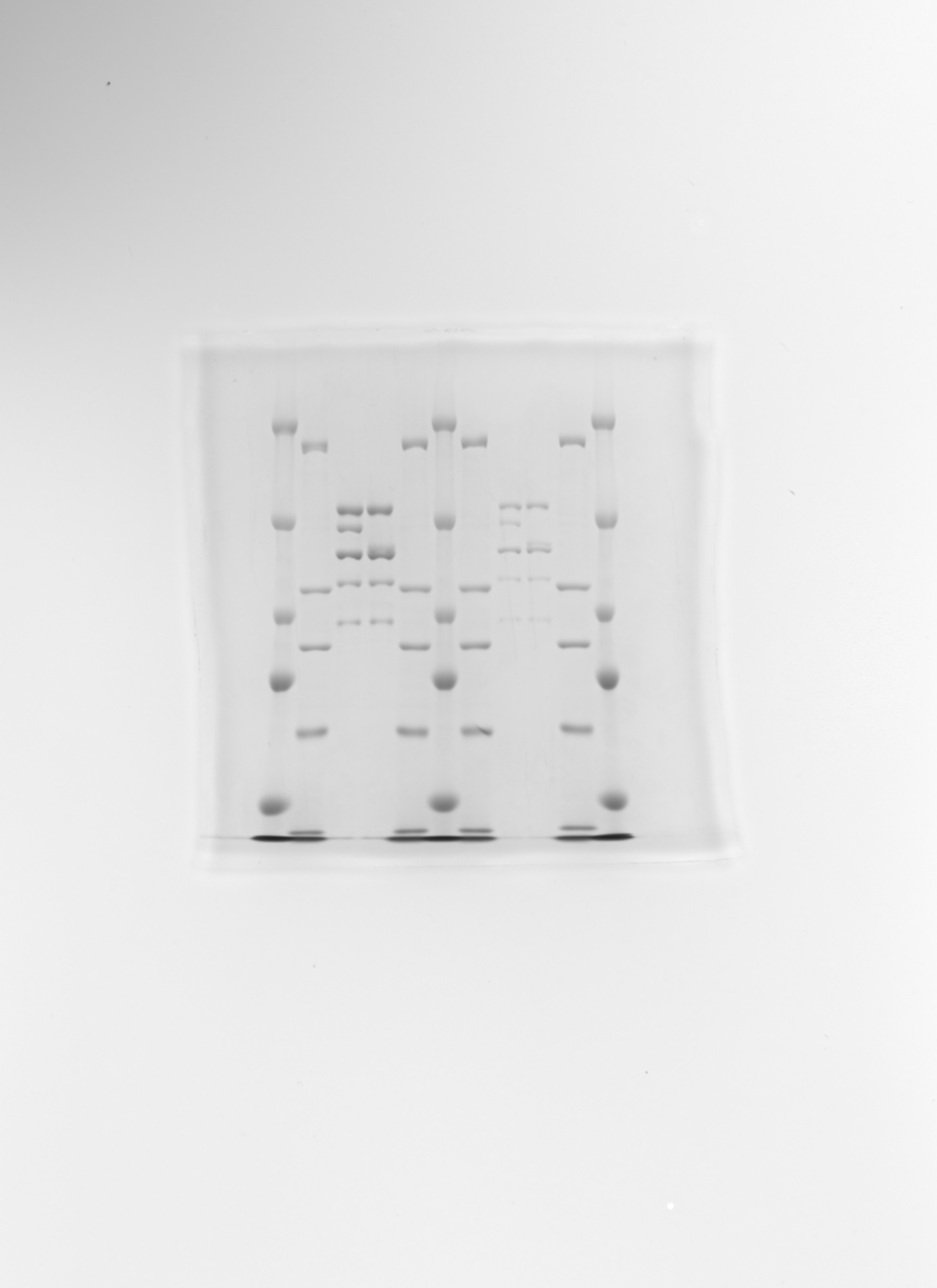

Supplement: Figure 4—figure supplement 3—source data 1. [file elife-78984-fig4-figsupp3-data1.zip › Figure 4-figure supplement 3-source data 1/Figure 4-figure supplement 3B-source data 1.tif]

Figure 5–figure supplement 1–source data 1

A

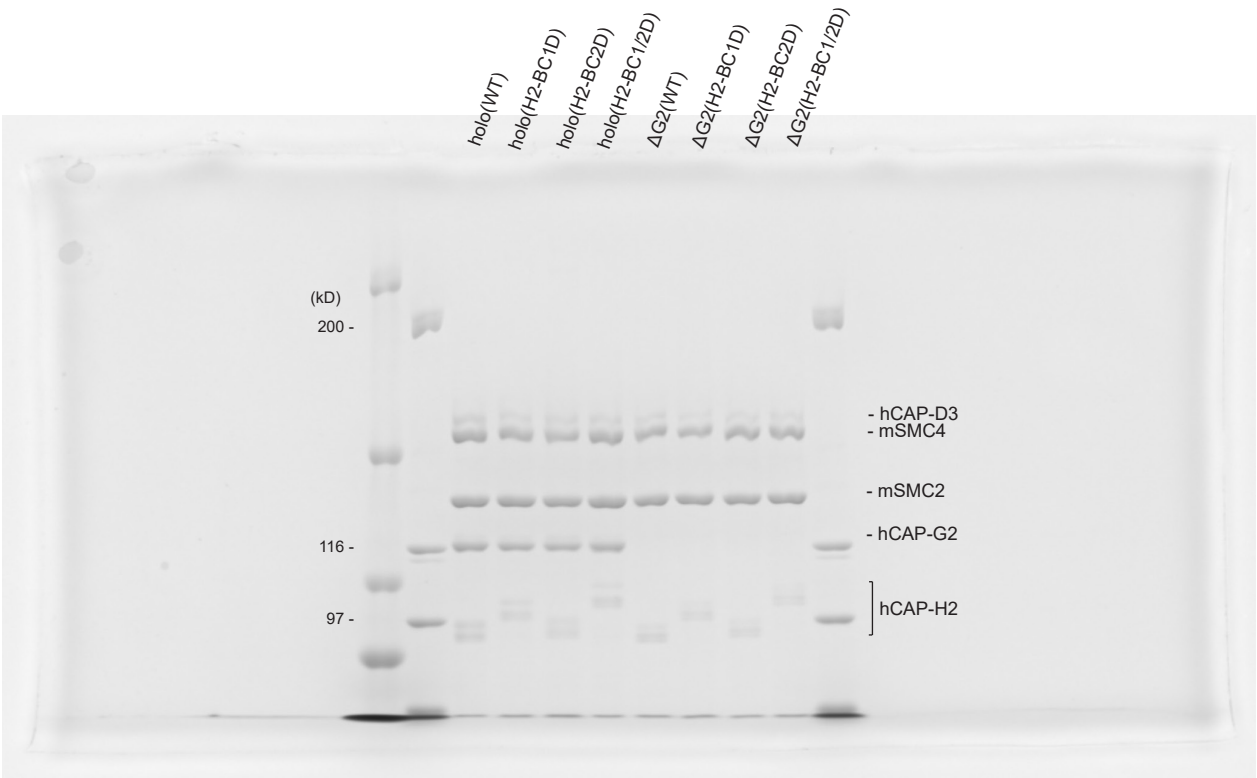

Supplement: Figure 5—figure supplement 1—source data 1. [file elife-78984-fig5-figsupp1-data1.zip › Figure 5-figure supplement 1-source data 1/Figure 5-figure supplement 1-source data 1.pdf]

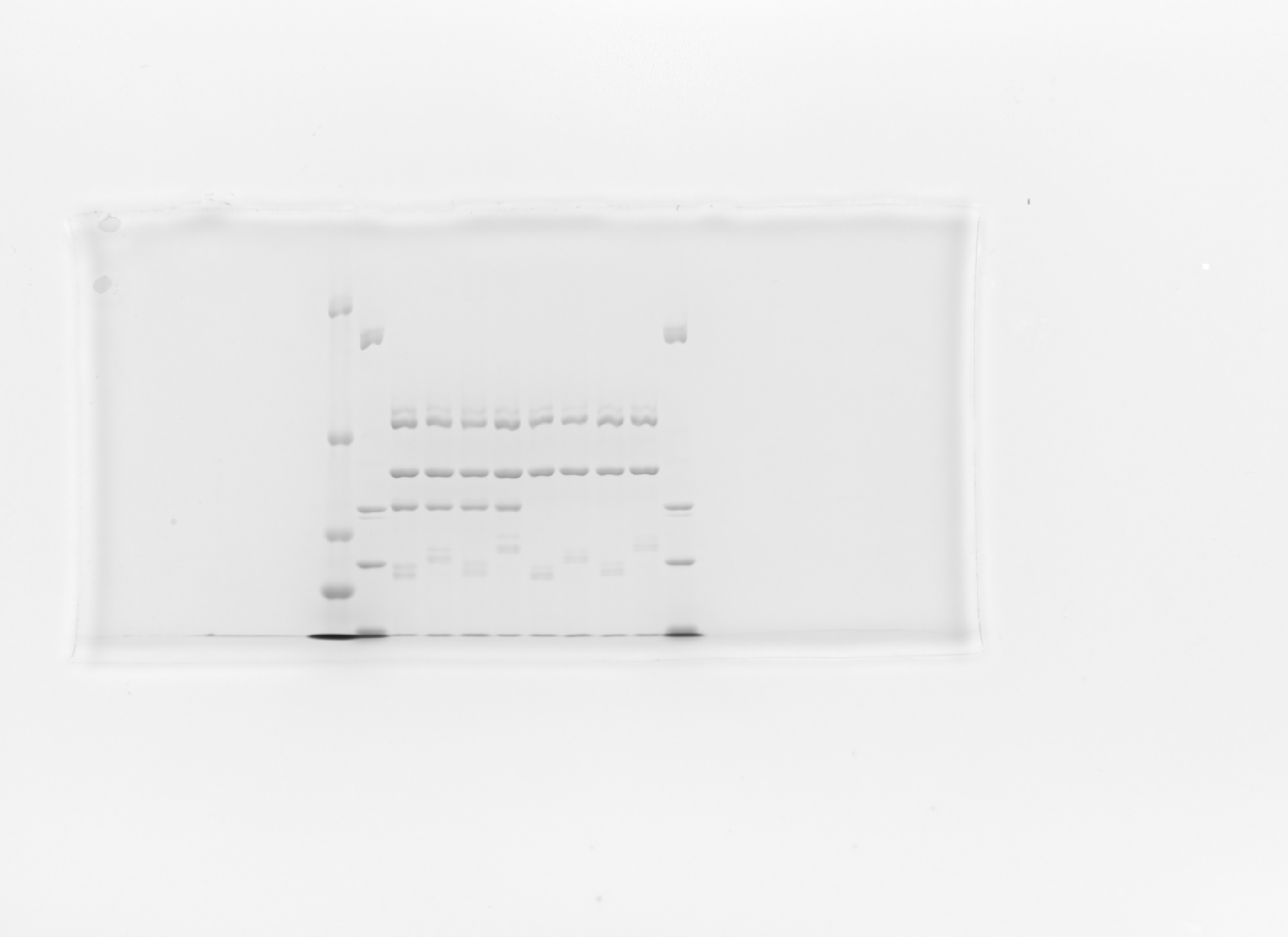

Supplement: Figure 5—figure supplement 1—source data 1. [file elife-78984-fig5-figsupp1-data1.zip › Figure 5-figure supplement 1-source data 1/Figure 5-figure supplement 1A-source data 1.tif]
