# Supplementary material for: Molecular dissection of condensin II-mediated chromosome assembly using in vitro assays
Source: eLife. 2022 Aug 19;11:e78984. doi: 10.7554/eLife.78984 (PMC9433093; doi:10.7554/eLife.78984)
Supplement: Figure 4—figure supplement 1—source data 1. [file elife-78984-fig4-figsupp1-data1.zip › Figure 4-figure supplement 1-source data 1/Figure 4-figure supplement 1-source data 1.pdf]

Figure 4–figure supplement 1B–source data 1

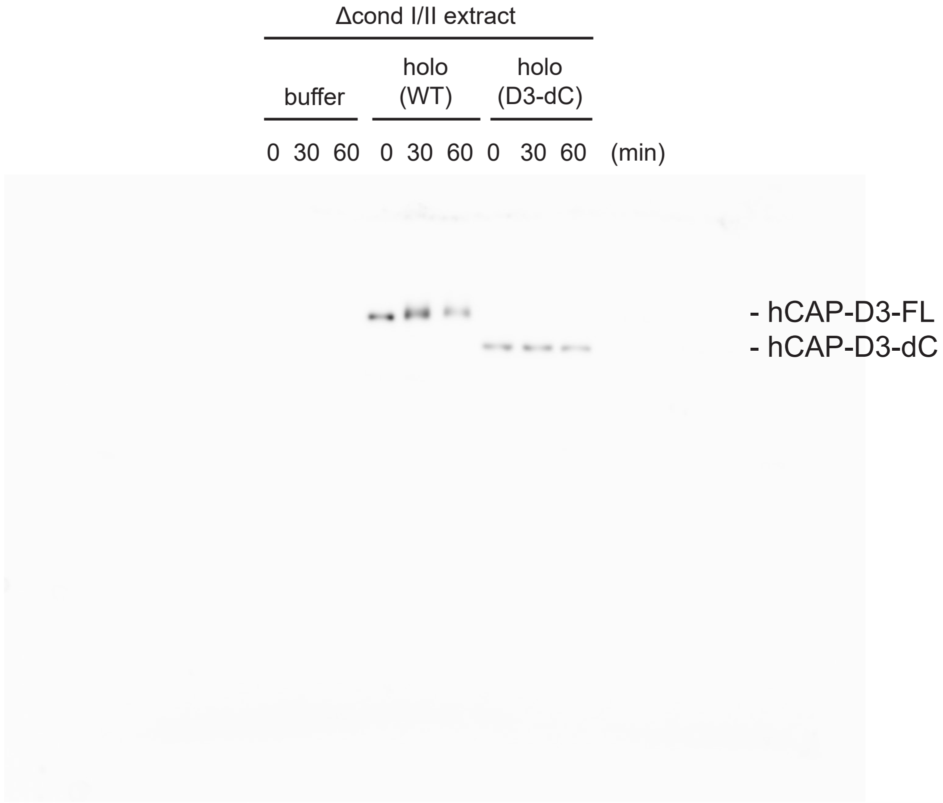

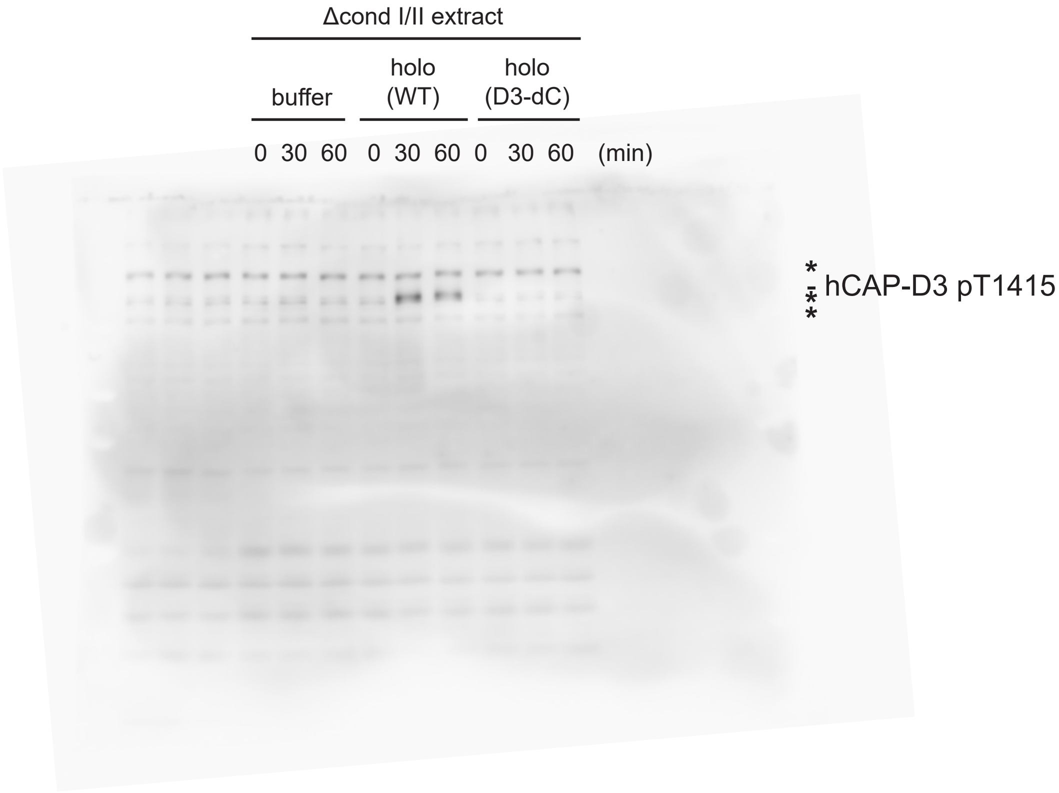

Δcond I/II extract

buffer | holo (WT) | holo (D3-dC)

0 30 60 0 30 60 0 30 60 (min)

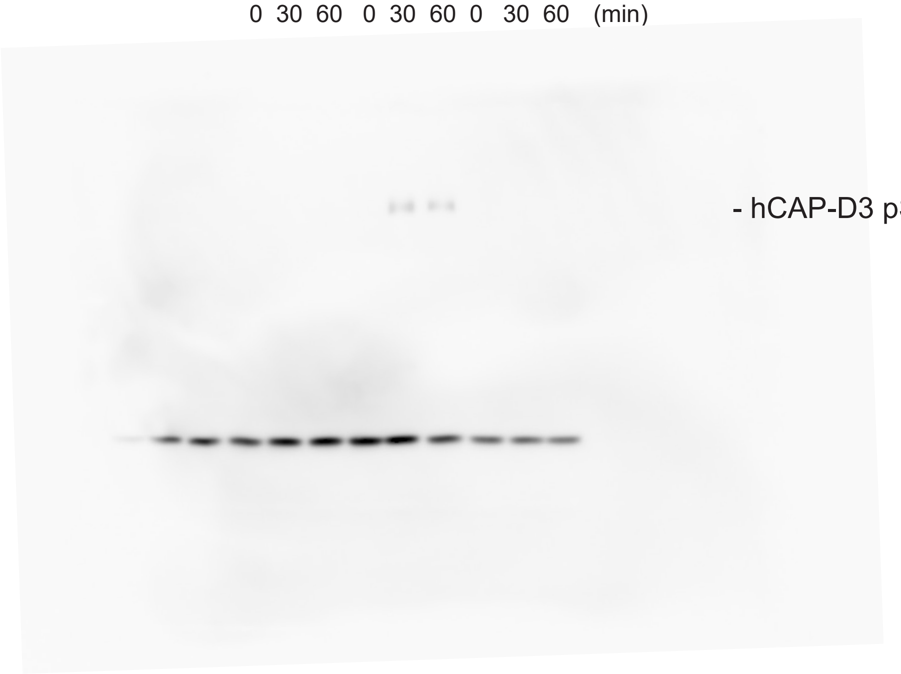

- hCAP-D3 pS1474

Δcond I/II extract

buffer | holo (WT) | holo (D3-dC)

0 30 60 0 30 60 0 30 60 (min)

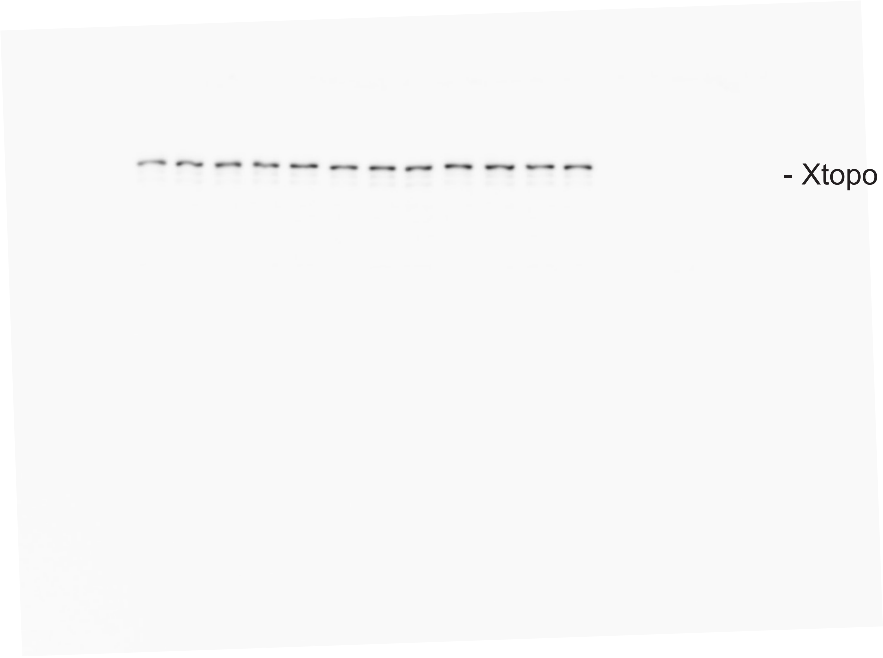

- Xtopo IIα
